# 3D Printing of Bioactive Gel-like Double Emulsion into a Biocompatible Hierarchical Macroporous Self-Lubricating Scaffold for 3D Cell Culture

**DOI:** 10.1021/acsami.3c12078

**Published:** 2023-10-12

**Authors:** Mahdiyar Shahbazi, Henry Jäger, Adeleh Mohammadi, Peyman Asghartabar Kashi, Jianshe Chen, Rammile Ettelaie

**Affiliations:** † Institute of Food Technology, University of Natural Resources and Life Sciences (BOKU), Muthgasse 18, 1190 Vienna, Austria; ‡ Faculty of Food Science and Technology, Gorgan University of Agricultural Sciences and Natural Resources, Gorgan 4913815739, Iran; § Faculty of Biosystem, College of Agricultural and Natural Resources, Tehran University, 31587-77871 Karaj, Iran; ∥ Food Oral Processing Laboratory, School of Food Science & Biotechnology, Zhejiang Gongshang University, Hangzhou 310018, China; ⊥ Food Colloids and Bioprocessing Group, School of Food Science and Nutrition, University of Leeds, Leeds LS2 9JT, U.K.

**Keywords:** double emulsion, LAOS, porous structure, printability index: tribology, cell proliferation

## Abstract

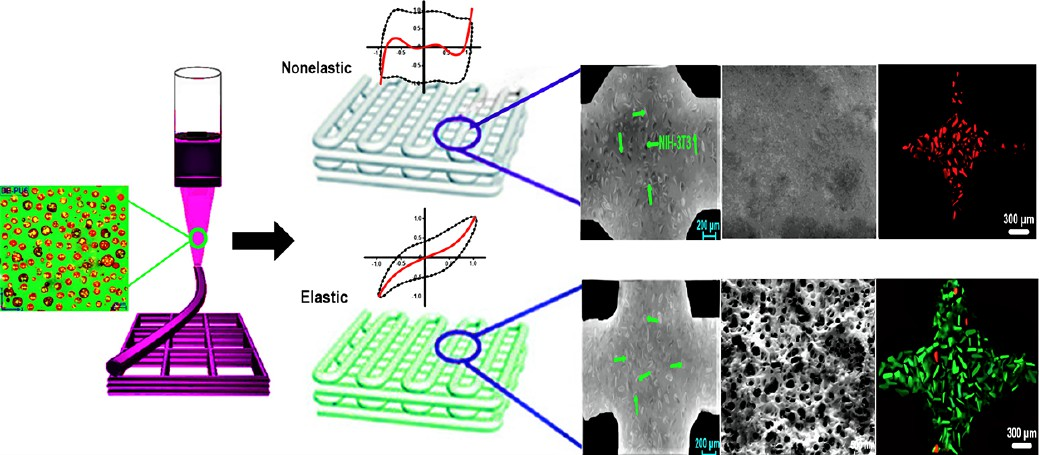

The interconnected hierarchically porous structures are of key importance for potential applications as substrates for drug delivery, cell culture, and bioscaffolds, ensuring cell adhesion and sufficient diffusion of metabolites and nutrients. Here, encapsulation of a vitamin C-loaded gel-like double emulsion using a hydrophobic emulsifier and soy particles was performed to develop a bioactive bioink for 3D printing of highly porous scaffolds with enhanced cell biocompatibility. The produced double emulsions suggested a mechanical strength with the range of elastic moduli of soft tissues possessing a thixotropic feature and recoverable matrix. The outstanding flow behavior and viscoelasticity broaden the potential of gel-like double emulsion to engineer 3D scaffolds, in which 3D constructs showed a high level of porosity and excellent shape fidelity with antiwearing and self-lubricating properties. Investigation of cell viability and proliferation using fibroblasts (NIH-3T3) within vitamin C-loaded gel-like bioinks revealed that printed 3D scaffolds offered brilliant biocompatibility and cell adhesion. Compared to scaffolds without encapsulated vitamin C, 3D scaffolds containing vitamin C showed higher cell viability after 1 week of cell proliferation. This work represented a systematic investigation of hierarchical self-assembly in double emulsions and offered insights into mechanisms that control microstructure within supramolecular structures, which could be instructive for the design of advanced functional tissues.

## 1 Introduction

The fabrication of next-generation engineering scaffolds with improved multifunctionality needs the rational design of structured materials, supported by an understanding of basic structure–function relations. To fabricate a well-defined hierarchically macroporous structure, bottom-up methods related to the self-assembly routes can be also employed.^1−3^ The adaptability of the mentioned processing techniques along with controlling the pore diameters can be an important challenge that remains the subjects of ongoing research studies. Of great interest in science and technology is the implementation of such porous hierarchies in artificial materials from the molecular level to the macroscopic dimensions with the highest possible precision. For example, a micropore structure (several to tens of microns) simplifies cell migration and proliferation, intracellular signaling, and cell adhesion.^4−6^ To produce innovative methods to design functional hierarchically structured porous materials, knowledge of the relationships among natural porous constructions and their functionalities is crucial. A wide variety of additive manufacturing techniques have been utilized to fabricate a hierarchically macroporous structure with well-defined porosity,^7−9^ providing an extensive selection in formulating biomaterials to offer outstanding bioactivity, biodegradability, biocompatibility, drug delivery, and endow suitable tensile strength.

Additive manufacturing, frequently known as 3D printing, is an innovative concept with valuable possibility to manufacture hierarchical mesostructures using specific control of printing inks, which can open up a new avenue to create efficient shape-changing objects.^10−12^ The 3D printing is widely utilized to produce anisotropic and microfibrous structures comparable to classical muscular tissue through an exclusive bottom-up layer-by-layer method.^13−15^ The printed microfibrous tubular structures have been employed in numerous vital human tissues including blood vessels, nerve ducts, and muscle fibers. They can efficiently encapsulate microfibrous structures because of their effective nutrient diffusion and bionic geometry.^16−18^ Numerous kinds of microfibrous structures can be produced through 3D printing, including flat microfibers,^18^ porous microfibers,^19^ coaxial/parallel laminar composite microfibers,^20,21^ and microfibers with microstructure patterns.^22^ The rheological parameters strongly influence the development of functionalized microfibrous structures with regards to actual extrudability,^21^ printability,^22^ printing accuracy,^23^ and shape fidelity^24^ of 3D-printed structures. Alongside this, accurate stability and formation of the printed objects are also essential to engineering a self-supported 3D architecture.^25,26^ A good understanding of the flow behavior,^27^ viscoelasticity,^28^ and thixotropic feature^29^ of printing inks are therefore necessary to develop a high-quality 3D object.

Conventionally there has been a correlation between ideal printable ink and the 3D printing quality of microfibrous structures with a porous structure.^30−32^ The lack of printability and shape fidelity rationally show inadequate mechanical strength/toughness postprinting with weak biocompatibility and bioactivity features. Therefore, 3D-printed microfibrous structures should offer the necessities of biocompatibility in addition to biodegradability to mimic and support cell growth and degrade according to the degree to which new cells develop. There is normally a positive correlation between biocompatibility and mechanical strength or toughness to uphold mechanical support concerning healing and inhibiting a stress-shielding impact. Principally, the comparatively poor biocompatibility relates to low mechanical strength and toughness,^33^ which can result in critical limitations during tissue engineering. For instance, the biocompatibility of microfibrous structures reduces after the incorporation of synthetic polymers into a high-toughness hydrogel.^34^ Hence, this evidence demonstrates serious issue in manufacturing fibrous hydrogels with a porous structure, improved toughness, high mechanical properties, and biocompatibility. Indeed, several extrinsic functionalizations on the scaffold structure were proposed to aid in improving their bioperformance,^35^ including encapsulation,^36^ biopolymeric additives,^37^ and dopants/coatings.^38^ This allows their appropriateness for tissue engineering, along with other biomedical applications. These tough microfibrous offer great biocompatibility to improved differentiation and proliferation of the human mesenchymal stem.^39^

However, more advanced materials should be utilized to strengthen printing inks to produce microfibrous and anisotropic structures,^40^ which proposes to uphold the printing architectures and be capable of sticking to the previously deposited layers. Concerning the signs of progress in the application of Pickering systems, the gel-like emulsions produced by Pickering emulsions are of evolving attention in 3D printing and bioprinting applications.^41,42^ As a semisolid colloidal dispersion, Pickering emulsion gels are stabilized by an adsorbed layer of solid particles, which combine the properties of both emulsions and gels.^43^ They offer crucial consistency in the 3D-printed structures, attributing to porous architectures.^44,45^ In recent years, there has been attention to exploiting emulsion gels as printable inks in the 3D printing process. For instance, Yu et al.^46^ investigated the 3D printing performance and freeze–thaw stability of soy-based emulsion gel ink as affected by varying percentages of guar and xanthan gums. Li et al.^47^ prepared an emulsion gel by self-assembly of gelatin and Pickering emulsions based on gallic acid modified-chitosan nanoparticles. Shahbazi et al.^48^ produced emulsion gels by oil replacement with diverse biosurfactants. The obtained emulsion gels exhibited good 3D printing performances with shear-thinning, thixotropic, and viscoelastic properties, demonstrating the potential to create a hierarchically porous structure using emulsion gels. Soy protein possesses optimal functional and physicochemical features to produce emulsion gels,^49^ which can be effectively printed to develop a well-defined 3D porous structure.^50,51^

The emulsion templating methods offer a relatively good environment for cells to proliferate, yet the poor mechanical properties and surface activity of common emulsifiers restrict their applications. Accordingly, the tailored functionalization of emulsion-based inks for bioprinting still remains a challenge, avoiding performance improvements in printing applications.^37^ Thus, the production of innovative bioinks with greater cytocompatibility and printing performance is required. The gel-like double emulsions offer a superlative candidate for housing cells as their functional properties endow to mimic the important fundamentals of native extracellular matrix (ECM),^52−55^ owing to the fact that an extremely swollen network can be obtained possessing excellent mechanical strength^52,56^ and self-lubricating property^57,58^ corresponding those of soft tissues. Additionally, the composition of gel-like double emulsions has been reported to be effortlessly tuned, revealing biological multifunctionality to several polymeric emulsifiers. This offers an effective environment for proliferation and cell adhesion.^59−61^

Concerning the bioactive properties, the application of high temperatures and extrusion force during 3D printing (or other processing conditions or even storage) can lead to thermal and/or other environmentally related degradation of bioactive compounds, thus decreasing their functional properties. Because of this, vitamin C is more susceptible to degradation because of its poor thermal stability.^54^ To overcome this limitation, the encapsulation of micronutrients and bioactive materials has attracted attention, which can help to reduce vitamin C degradation. High-intensity technology is a high-power (>1 W cm^–2^) and low-frequency (20–100 kHz) ultrasound process also known as “power ultrasound” or “high-intensity ultrasound” (HIU) for the encapsulation of bioactive compounds. HIU emulsification also offers a fast and simple yet efficient procedure, by which a double emulsion is likely developed through low amounts of surface-active materials.

Herein, we hypothesized that the utilization of a double emulsion gel-like structure in an extrusion-based 3D printing method offers the production of macroporous 3D structures with outstanding cell biocompatibility and improved printing quality because of the degree of control over the pore diameters in the artificial materials. Accordingly, a double emulsion of water-in-oil-in-water (W_1_/O/W_2_) containing vitamin C was prepared with a hydrophobic emulsifier and soy protein particles. After rheological and mechanical characterizations, the precursor double emulsion-based inks were printed via an extrusion-based 3D printing system to fabricate a hierarchical porous gel-like structure. Furthermore, we prepared bioactive 3D-printed scaffolds and comprehensively characterized their antiwearing, self-lubricating, and mechanical properties. Finally, bioactive double emulsion-based bioink prepared with NIH 3T3 cells was used to prepare 3D-printed scaffolds, and the cell response and survival of cells in different biological environments were assessed.

## 2 Methods and Materials

### 2.1 Materials

The soy protein isolate (SPI) (moisture: 4.83%, fat: 0.32%, protein: 92.88%, ash: 3.40%, pH: 7.09, and viscosity of 1 wt % solution: 10.0 cP) was obtained from Archer Daniels Midland Company (ADM, Decatur, IL). Vitamin C (cobalamin) was purchased from Pharmanostra, China, Hong Kong. Polyglycerol polyricinoleate (Grindsted PGPR 90) was provided by Danisco Canada Inc., Scarborough, Ontario, Canada. Sunflower oil (density equal to 0.92 g cm^–3^ at 20 °C) was prepared from the local market. All other reagents were of analytical grade.

### 2.2 Preparation of W_1_/O Primary Emulsions

The primary (W_1_/O) emulsions (Supporting Information, Section S1) were prepared as the first step in the formation of double emulsions. Vitamin C was added to the internal aqueous phase at 75 mg/mL (w/v), which is based on the daily recommended vitamin C.^62^ This concentration was also selected to simplify the spectrophotometric detection of vitamin C.^63^ The oil phase (O) consisted of sunflower oil containing 5% (w/w) PGPR 90 as a hydrophobic emulsifier. The aqueous and oil phases were both heated to 45 °C. Water and oil phases were then cooled to room temperature, and vitamin C was added to the water phase, where the solution was stirred vigorously in the dark to make it completely dissolve. The W_1_/O primary emulsion was prepared by adding the inner aqueous phase (W_1_) (0.2 mass fraction) to the oil phase (0.8 mass fraction). The mixture was pre-emulsified through a rotor-stator device (SilentCrusher M, Heidolph, Germany) at 12,000 rpm for 2 min. An additional homogenization was tested to break down the clusters produced at a high homogenization shearing rate (12,000 rpm for 1 min).^50^ The mixture (70 mL each) was poured into a glass double-walled beaker fitted with a cooling installation. Then, a high-intensity emulsification process (VC 750, Sonics & Materials, Inc., CT) fitted with a 13 mm diameter probe was used in the ultrasonic processing of W_1_/O emulsions. The W_1_/O emulsions were subjected to ultrasound treatments (frequency 20 kHz; amplitude 60%; power 450 W) for 0, 2, 4, 6, and 8 min (with pulse mode durations of 2 s on and 4 s off), respectively. The untreated samples were used as the control and stored at 4 °C. During the ultrasound, the probe was immersed in the emulsions to a depth of 25 mm, and ice-cold water was cycled around the glass double-walled beaker. The sample temperature was maintained below 8 °C. The ultrasound intensity was 30.09 ± 1.24 W cm^2^ as measured referring to a protocol by previous work.^64^ The W_1_/O primary emulsions were signed as PU-2, PU-4, PU-6, and PU-8, which proposed the treated emulsion with sonication times of 2, 4, 6, and 8, respectively. Control emulsion (PU-0) was the W_1_/O primary emulsion without the sonication treatment. Normally, the emulsion type formed can be determined via two tests, namely, the dilution test (Supporting Information, Section S1) and the electrical conductivity. In the dilution test, when the emulsion is diluted with water (continuous phase) and stays stable, it is an O/W emulsion, but if it gets destabilized, it is a W/O emulsion. In the case of electrical conductivity test of the emulsion samples, it was determined and measured through a four-point probe technique with a Keithley Source Meter (6517 A, Cleveland, OH) at ambient conditions.

### 2.3 Preparation of W_1_/O/W_2_ Double Emulsions

Double emulsions were produced in SPI dispersion (18%; pH 6.8; containing 0.1 m NaCl) and preheated to 45 °C. The W_1_/O primary emulsion (0.30 mass fraction) was added to the external aqueous phase (W_2_) (0.70 mass fraction) stabilized by soy protein particles (Supporting Information, Sections S2 and S3). The mixture was emulsified using a rotor-stator device (SilentCrusher M, Heidolph, Germany) at 12,000 rpm for 2 min. The double emulsions, W_1_/O/W_2_, were stored at 4 °C for further analysis and testing (Supporting Information, Section S4). The codes of DE-PU0, DE-PU2, DE-PU4, DE-PU6, and DE-PU8 were considered for the double emulsions containing PU-0, PU-2, PU-4, PU-6, and PU-8 primary emulsions.

### 2.4 Characterization of Emulsions

#### 2.4.1 Droplet Size and Electrical Charges of Emulsions

The inks were diluted to a droplet level of about 0.005 wt % with deionized water at the pH of emulsions (pH = 6.8). The dispersion was stirred gently at room temperature to ensure the emulsions were homogeneous. The droplet sizes and particle size distribution (PSD) of the inks were measured with a laser diffraction device (MS2000, Malvern Instruments Ltd., Worcestershire, UK) for 14 days. The device measured the size based on the scattering of a monochromatic beam of laser light (*λ* = 632.8 nm). The droplet size was specified as the surface-weighted mean (*d*_3,2_) = *(∑n*_*i*_*d*_*i*_^3^ / *∑n*_*i*_*d*_*i*_^2^), where *n* is the number of droplets with a diameter *d*.^65^ The electric potential (ζ- potential) of the printable inks were also obtained through a Zetasizer Nano-ZS90 (Malvern Instruments, Worcestershire, UK) at a fixed detector angle of 90°. To minimize multiple scattering effects, the emulsions were diluted to a final concentration of 0.005 wt % with deionized water before analysis. After loading the samples into the chamber of the Zetasizer, they were equilibrated for 5 min before zeta potential data were obtained over 40 continuous readings.

#### 2.4.2 Confocal Laser Scanning Microscopy (CLSM)

CLSM images of the emulsions were taken with a Nikon Eclipse Ti inverted microscope (Nikon, Japan). A portion (5 mL) of the inks was stained with the appropriate amount of Nile Blue A (1.0%, w/v) in deionized water or the blend of Nile Blue A (1%, w/v) and Nile Red (0.1%, w/v) in 1,2-propanediol (including deionized water, 20 μL g^–1^) to mark the protein and/or modified MMC and oil droplet, respectively. The level of both Nile blue A and Nile red solutions was 0.01% (w/v). The excitation wavelengths of the fluorescent in the system were 488 nm (Nile red) and 633 nm (Nile blue A). The ink microstructures were imaged at ambient temperature directly after staining. All images were obtained at 40× magnification and processed using Olympus Fluoview software (version 2.1, Olympus, Tokyo, Japan).^65^

#### 2.4.3 Rheological Tests

A HAAKE MARS III modular advanced rheometer system (Thermo Scientific Co., Ltd., Waltham, MA) was used to monitor the rheological properties of double emulsions. The rheological behavior of ink samples was characterized by an AR 2000ex rheometer (TA Instruments, New Castle, DE) using parallel plate geometry (diameter 40 mm, gap 1 mm). To evaluate the steady rheological properties, the shear stress (τ) was measured as a function of increasing shear rate (γ̇) from 0.1 to 1000 s^–1^.

It was coupled with a parallel plate probe P35TiL with a gap of 1 mm. To determine the linear viscoelastic region (LVR), a stress sweep test (τ, 10^–1^–10^2^ Pa) was primarily performed at a constant frequency of 10 rad s^–1^ to detect the corresponding elastic (*G′*) and loss moduli (*G″*). Moreover, the impact of the shear rate (0.1–10^3^ s^–1^) on the apparent viscosity of the double emulsion was evaluated.^66^

Finally, a five-interval thixotropy test (5-ITT) was used to gather thixotropic data for the double emulsions. The 5-ITT detected the viscosity profiles of the samples under alternating high and low shear rates (80 or 0.1 s^–1^) for 500 and 510 s, respectively.

#### 2.4.4 Lissajous-Bowditch Curves

The construct of Lissajous plots obtained from large amplitude oscillatory shear (LAOS) analysis and the Chebyshev coefficients were conducted following our previous study.^65^ Based on the Fourier spectrum (the signal-to-noise ratio, or S/N, was estimated at the range from 10^3^ to 10^5^, at the strains of 1.1, 6.1, 61, and 200%, respectively) and a Chebyshev polynomial-based stress decomposition method, the torque-deformation waveform data were performed using MITLaos program (Version 2.2 beta). To analyze the nonlinear response of samples, the torque-deformation waveform data at different strains (1.1, 6.1, 61, and 200%) and frequencies (1 and 10 rad/s) were collected using a HAAKE MARS60 rheometer (HAAKE Co., Germany) with native rheometer control software (Rheowin Job Manager). The raw strain–stress data were collected at a sampling rate of 512 s^–1^. The S/N is the ratio of the amplitude of the highest peak (the first harmonic) divided by the standard deviation of the noise.

### 2.5 Printing Process

The machine architecture is very simple, where the extrusion head transfers in the *XZ* plane construction while the platform translates along the *Y*-axis (Supporting Information, Section S5). For the 3D printing process, a snowflake (80 mm diameter, 10 mm height), octopus (80 mm diameter, 40 mm height), and cylindrical (40 mm diameter, 60 mm height) were initially modeled and converted to STL files (SolidWorks, Dassault Systèmes, SolidWorks Co., Vélizy-Villacoublay, France). A nozzle with an inner diameter of 1 mm was employed to extrude emulsion gels on a silicon platform using a direct-ink-write 3D printing process. After comprehensive consideration, emulsions with improved flow behavior were designated for the printing process, with layer height being 0.5 mm, shell 2 mm, and nozzle movement speeds during printing being 20 mm s^–1^.^50^ The main printing process was carried out at ambient temperature.

### 2.6 Printability Index (*Pr*)

The ink printability has been associated with the ability to produce square-shaped internal pores in a printing object. A perfect square geometry of pores yields a value of 1 with the following printability index (*Pr*):
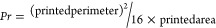
1

### 2.7 Microstructure of 3D-Printed Objects

A field-emission scanning electron microscope (FE-SEM, S-4700, Hitachi, Japan) was used to detect the morphological structure and integrity of the 3D-printed architectures. Before image processing, the samples were coated with a thin layer of gold at 20 mA for 2 min (JEOL JFC-1600, Auto Fine Coater, Tokyo, Japan). The applied energy levels were in the range of 5 kV to prevent the film samples from being damaged with a magnification of 20.00 kX.

### 2.8 Mechanical Strength of the 3D-Printed Objects

Mechanical assay for tensile strength of dumbbell-shaped 3D structures (10 mm gauge length, 2 mm width, and 2 mm thickness) was performed at 100 mm min^–1^ through an Instron 3366 electronic universal testing machine (Instron Corporation, MA). The elastic modulus (*E*) of 3D-printed samples was determined by the average slope over 10–30% of strain from the stress–strain curve. The fracture energy (Γ) and toughening mechanism of the 3D-printed objects were evaluated as follows. (1) Each loading–unloading cycle was applied to the 3D-printed constructs under a tensile strain lower than their corresponding yielding strains. (2) The successive and progressive stretches, where each specimen was stretched to different strains in the first loading and then relaxed to zero force, followed by the second loading. The *E*_second_/*E*_first_ and *Γ*_second_/*Γ*_first_ were determined and used to evaluate the effect of various stretches on the fracture process and toughening mechanism for the 3D structures. For the recovery experiments, the notched samples were tested by a cycle of loading–unloading at a fixed strain (*ε* = 400%). Then, the deformed and relaxed notched samples were sealed in a polyethylene bag and stored in a water bath at 90 °C. Finally, the specimens were taken out at different time intervals and cooled down to room temperature for tensile tests again.^50^

### 2.9 Tribology Measurements

Oscillatory tribology measurements were conducted on a commercial shear rheometer (MCR302, Anton Paar, Graz, Austria) equipped with a custom-made measuring head. The measuring head holds three probing pins. Three steel spheres (Kugel Pompel, Austria, stainless steel types 1.4404, *d* = 5 mm, surface roughness *S*_q_ < 0.2 μm) were used as a counter material (to mimic the hard palate in the oral cavity) for testing friction on the samples. The samples were fixed onto single-use measuring plates (EMS/TEK500/600, Anton Paar) using double-sided adhesive tape, and then mounted onto the bottom plate of the rheometer (P-PTD200/80-I, Anton Paar). The friction torque was recorded over a deflection angle range of 0° ≤ *φ* ≤ 16° at a sliding velocity of *v* = 1 mm/s. All measurements were performed in torque-controlled mode at a normal force of *F*_N_ = 0.2 N. They were also performed in triplicate without any lubricant. Each test lasted for 0.5 h and three repeat times were performed to evaluate the average wear volume and friction coefficient.

### 2.10 Statistical Analysis

Unless otherwise stated, all measurements were carried out in triplicate. One-way analysis of variance (ANOVA) with a 95% confidence interval was used to compare the significance of the results obtained. Statistical analysis was performed using SPSS software, version 19.0.

## 3 Results and Discussion

### 3.1 Characterization of Single and Double Emulsions

The development of a high-quality 3D-printed hierarchical porous structure is directly associated with the engineering of a printable emulsion with shear-thinning properties, viscoelastic features, and thixotropic behavior, requiring a deep understanding of materials’ printability and extrudability.^10^ Besides, the sizes of the primary and secondary emulsion droplets are important influential features to form a highly stable printable ink.^50^ In particular, the primary droplets (the inner aqueous phase containing vitamin C in the oil droplets stabilized by PGPR) should be small enough to offer encapsulation inside the secondary droplets (oil droplets stabilized by soy protein particles), which themselves must be enough to avoid creaming (Supporting Information, Sections S2 and S6). As the integration of a bioactive gel-like double emulsion into 3D printing to construct a printed hierarchical porous architecture has not yet been explored, a comprehensive characterization of the size of W_1_/O and secondary W_1_/O/W_2_ double emulsions, as well as their interfacial framework and flow behavior, was evaluated as a function of HIU time processing.

#### 3.1.1 Primary W_1_/O Emulsion

The formation of W/O emulsion can be proved through a simple dilution measurement and electrical conductivity of the emulsion system.^48^ When oil is in the continuous phase, if water is introduced, it will not mix with the W/O emulsion, but the incorporation of oil will dilute the emulsion with a full dissolution or blurred edge. As can be seen (Supporting Information, Section S1), the oil added to the emulsion showed an obvious edge, verifying a W/O-type emulsion. Additionally, we performed an electrical conductivity test to further verify the type of emulsion system. The key idea behind the electrical conductivity test is that water is a good conductor of electricity but oil is not. Hence, if the emulsion sample conducts electricity, it is an O/W emulsion, but if it does not, it is a W/O emulsion. The conductivity values of all primary emulsions were low and ranged from 0.06 to 1.02 μS/cm. These results confirm that the system belongs to a W/O-type emulsion.

**Figure 1:**
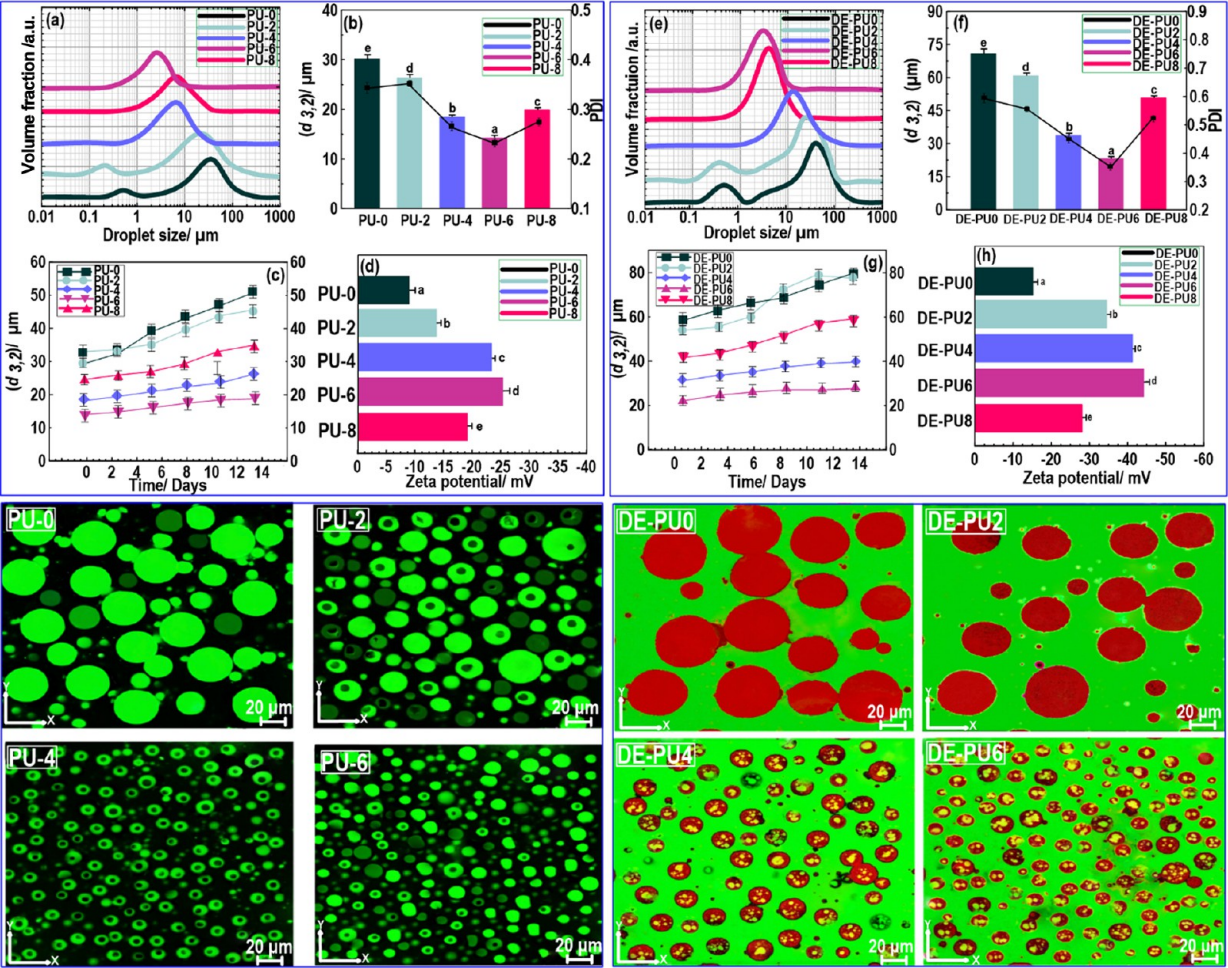
Up-left: (a) particle size distribution, (b, c) droplet size, and (d) ζ-potential of primary emulsions. Down-left: CLSM image of primary emulsions. Up-right: (e) particle size distribution, (f, g) droplet size, and ζ-potential (h) of double emulsions. Down-right: CLSM image of the double emulsions.

Controlling the particle size is an effective way to meet the functional requirements of printable inks for 3D printing purposes. It was stated that a reduction in the particle size of ink dispersions improves the ink functionality in terms of printability and shape fidelity^37^ or construction of a porous structure.^40^ The effect of ultrasound conditions (frequency 20 kHz; amplitude 60%; power 450 W for 0–8 min) on the diameter size of primary aqueous droplets of W_1_/O emulsion (containing 75 mg/mL (w/v) vitamin C in the internal aqueous phase) was assessed using light scattering (Figure 1, up-left) and CLSM (Figure 1, down-left) measurements. Varying the ultrasound time controls the intensity of cavitation and the size of the resultant emulsion droplets.^50^ The W_1_/O emulsions were fabricated with the same PGPR concentration (5%, w/w) but different HIU times. This PGPR was used as it was the minimum amount required to create stable multiple emulsions.^65^ To develop a stable double emulsion with improved encapsulation efficiency, small droplet size and resistance against coalescence are necessary.^65^ The PSD and mean droplet size (*d*_3,2_) of the W_1_/O emulsions, stabilized by PGPR, are presented in Figure 1a,b, respectively. The primary emulsion droplets with no sonication process (PU-0) presented a bimodal size distribution profile with a (*d*_3,2_) value of around 30 μm. Compared to PU-0, PU-2 showed a lower PSD with a tendency for exhibiting a bimodal distribution (Figure 1a) but yet a comparable polydispersity index (PDI) (Figure 1b). However, its (*d*_3,2_) value was slightly decreased to around 26 μm, which may be attributed to the ultrasound effect.^61^ In contrast, applying HIU treatment with higher time produced a W_1_/O emulsion with monomodal distribution, and also shifted the peak of PSD to the lower sizes (Figure 1a). Compared with PU-0 and PU-2, the mean particle size (*d*_3,2_) of PU-4, PU-6, and PU-8 was decreased (Figure 1b), which was stable against coalescence for 14 days (Figure 1c). This is especially a case at the HIU time output of 6 min (*p* < 0.05), which resulted in the lowest PDI (Figure 1b). This indicates the highest particle homogeneity among all of the tested samples. These results illustrate a better emulsification effect of PGPR as a result of HIU treatment, which effectively decreased the size of primary aqueous droplets and developed a well-defined W_1_/O emulsion. It is well-known that the sonication time improves the emulsification properties due to the cavitation phenomenon.^45^ Increasing the sonication time results in greater cavitation and shearing forces, causing more disruption of emulsion droplets and reduced retention of internalized aqueous phase droplets. It should be noted that the PU-8 emulsion showed bigger droplet sizes than those of DE-PU4 and DE-PU6 (Figure 1b). As the HIU time reached 8 min, there was the development of an even smaller droplet size in the “freshly” prepared PU-8 emulsion compared to PU-4 and PU-6 (data not shown). Therefore, when the W/O interface is formed, the level of PGPR is likely inadequate to adsorb at the W_1_/O of these newly developed droplets before coalescence, which could result in a relatively larger droplet size. It is also worth mentioning that we did not produce emulsions with the application of more than 8 min sonication time since the aqueous phase was too viscous, which was not suitable for the 3D printing process (data not shown).

To further explore the relation between the particle size dispersal and emulsion microstructure, the interfacial framework and microstructure of the W_1_/O emulsion processed by HIU were detected by CLSM (Figure 1, down-left). The droplets detected to lack the entrapped aqueous phase might be in a different focal plane. In the absence of HIU treatment (PU-0 sample), the droplet size was relatively large with a solid and orderly interface layer. This shows that the addition of PGPR, followed by shearing force (with no sonication process), slightly contributed to the development of a typical W/O emulsion (Supporting Information, Section S1). Generally, there are two kinds of attractive droplet−droplet interactions induced by polymeric or particulate type surfactant-stabilized emulsions, which are depletion flocculation and bridging flocculation.^48^ Once the surfactant is unadsorbed or poorly adsorbed, depletion flocculation can be driven by the osmotic pressure gradient related to surfactant exclusion from a narrow area adjacent to the droplets. This leads to the droplet’s attraction toward each other.^48^ In contrast, adsorbing of a lower level of surfactant onto the droplet surface results in the droplet’s linkage via bridges and consequently their flocculation.^48^ The CLSM micrograph of the PU-2 emulsion also shows the development of large particle size in the continuous phase with rather uneven size distribution, with no evidence of local flocculation. This tendency could be due to a reduced surface hydrophobicity and structural flexibility of PGPR due to insufficient emulsification treatment. This proposes a decreased specific surface area upon low-period HIU processing. This change was not favorable for diffusion to the expansion and border toward the superficial oil drops, which resulted in the sharing of a layer of surfactant between adjacent droplets, leading to phase separation (Supporting Information, Section S4).^45^ With increasing HIU treatment time, it can be seen that droplets existed in the unflocculated and separated shapes, and the droplet size gradually reduced with increasing HIU treatment time. Once the treatment time reached 6 min (PU-6 sample), the emulsion droplets were the smallest, which was in accordance with the PSD and PDI results. Moreover, CLSM images of PU-4 and PU-6 clearly showed the attendance of the darkened areas inside the green fluorescent water droplets (Figure 1, down-left), which could specify an air void space.^40^ Because of the good surface activity of PGPR followed by an improvement in the emulsification process, PGPR could efficiently adsorb at the W/O interface and produce a dense interface skeleton. This was associated with the physicochemical process resulting from the effective sonication treatment, which led to more disruption of droplets and therefore reduced the droplet size.

The electrostatic forces between the emulsion droplets are the main factor in the stability of emulsions against flocculation. In this case, zeta (ζ) potential is the main parameter to monitor the physical stability of emulsion systems, which provides an indication of the electrostatic repulsion between droplets as they approach each other.^50^ Since it is associated with the surface charge of droplets, it can be applied to the prediction of the storage stability of emulsion-based inks to a certain extent.^50^ Commonly, a more stable system against droplet aggregation will tend to have a higher absolute ζ-potential value. Figure 1d shows the impact of the applied sonication time on the ζ-potential at the air-in-water interface of the W_1_/O system (please note that the negatively charged values are the ζ-potential at the air–water interface as some air bubbles were entrapped into the water as detected by CLSM in Figure 1, down-left). The ζ-potential of air dispersed in water with respect to PU-0 showed a comparatively low net-negative charge (about −10 ± 0.1 mV). As the HIU time increased, the net charge at the air–water interface presented a progressively increasing trend.^50^ The most negative value of −25.4 mV was found to occur for PU-6, which was produced by HIU at a processing time of 6 min (*p* < 0.05).

### 3.2 Characterization of W_1_/O/W_2_ Emulsions for 3D Printing

#### 3.2.1 Particle Size and Electrical Charge

Freshly produced primary W_1_/O emulsions were utilized in the preparation of the double emulsion-based inks (W_1_/O/W_2_) for the 3D printing process. For the following measurements, the double emulsions containing primary W_1_/O emulsions stabilized by soy protein particles were emulsified through a simple shearing force treatment with no sonication process. To verify the development of secondary oil droplets with sufficient size to encapsulate the primary aqueous droplets, PSD, (*d*_3,2_), PDI, and ζ-potential were also evaluated for the prepared W_1_/O/W_2_ emulsions (Figure 1, up-right). Unsurprisingly, the droplet sizes of double emulsions were notably larger than those of W_1_/O emulsions. Besides, there is a prominent difference in the droplet sizes of the double emulsion samples formulated by W_1_/O emulsions. Compared to the double emulsions containing HIU-treated W_1_/O emulsions, the W_1_/O/W_2_ emulsion including the nonsonicated W_1_/O sample (DE-PU0) showed a multimodal PSD (Figure 1e) with a (*d*_3,2_) of about 70 μm (Figure 1f). This effect is possibly associated with the development of droplet/protein aggregation with weaker physical/colloidal stability of the interfaces developed by the surfactant used (or even soy protein particles). A similar result was detected for the W_1_/O/W_2_ emulsion containing a 2 min-sonicated W_1_/O sample (DE-PU2), however, with a significantly lower (*d*_3,2_) (*p* < 0.05). Because of the comparatively bigger droplet diameter of DE-PU0 and DE-PU2 double emulsions, they were very unstable to gravitational separation. This provides an optically opaque (white) layer of the droplets, which was obviously noticeable on the top of the emulsions after a few storage hours (data not shown). In contrast, a reduction in the fraction of larger droplets and particles was detected for the double emulsions containing PU-4 and PU-6 W_1_/O emulsions (Figure 1f). Compared to DE-PU0 and DE-PU2, the (*d*_3,2_) of the DE-PU4 and DE-PU6 emulsions was also stable for 2 weeks (Figure 1g). This droplet size observation is consistent with other ultrasonic emulsification studies about the impact of HIU processing.^61^ In the current study, there is an important effect of the colloidal stability resulting from the presence of soy protein particles in DE-PU4 and DE-PU6. The soy protein particles improved the physical stability against droplet coalescence, resulting in the adsorption of particles at the interfaces and also their favorable flow behavior.^66^ It should be noted that the DE-PU8 emulsion showed a bigger droplet size with a physically unstable structure than those of DE-PU4 and DE-PU6. As mentioned above, this sample contains PU-8 W_1_/O emulsion having higher droplet size, whose diameters were altered appreciably upon storage likely forming aggregated droplets, which nonetheless can still be stable against coalescence (due to Pickering nature of the emulsions).^65^

**Figure 2:**
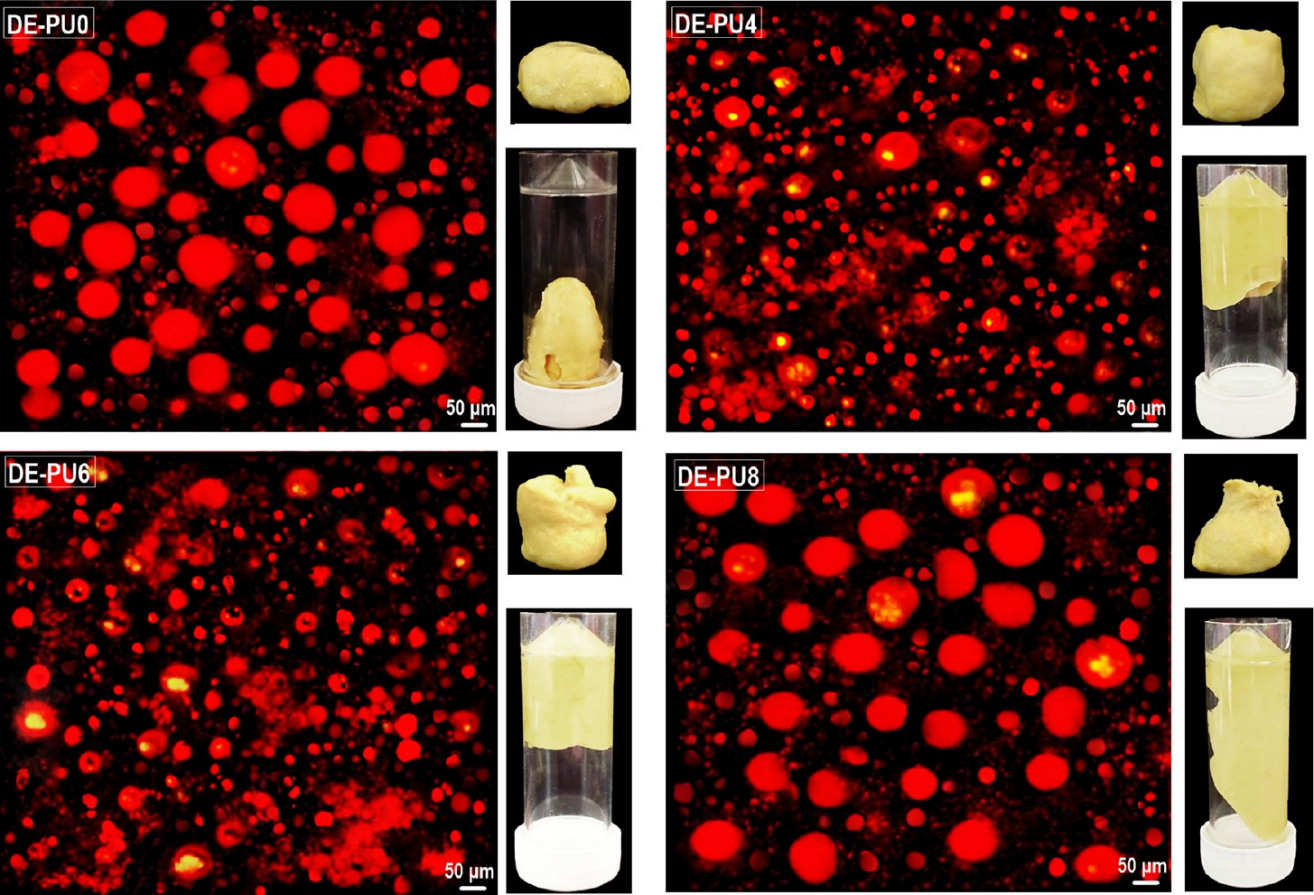
CLSM images of double emulsions with the visual observation of their relevant gel-like structure.

Thus, the physical stability of the DE-PU4 and DE-PU6 emulsions is likely related to the gradual particle adsorption at the O/W interface. Figure 2 presents that there is some droplet flocculation in the system. The interactions between the emulsion droplets and the interfacial layer affect the dominant flocculation mechanism. Based on Figure 2, the occurrence of droplet aggregation is clearly shown, especially for DE-PU4, DE-PU6, and DE-PU8 samples. The droplets attained the maximum amount of flocculation in the case of DE-PU6 emulsion. Considering the obtained data, it could be concluded that the droplet aggregation process is governed by direct van der Waals, as well as mediated bridging attraction between droplets on one hand, and the steric and electrostatic repulsion amongst them on the other.

As mentioned above, the stability of emulsion systems through evaluating the electrophoretic features of the particles/droplets can be characterized by ζ-potential. As a main physical parameter of double emulsion, the particles/droplets’ surface charge affects their solubility and electrostatic interactions with other particles/droplets.^48,65^Figure 1h shows the ζ-potential of different soy-based double emulsions containing HIU-treated W_1_/O emulsions. As expected,^50^ there was a greater decrease in the magnitude of ζ-potential for all double emulsions compared to primary W_1_/O emulsion droplets. This may be due to the presence of the anionic character of soy proteins.^65^ The DE-PU2 emulsion contained untreated primary emulsion droplets (PU-0) and showed a negative ζ-potential value (anionic feature) of about 12 ± 0.2 mV. This proposes that soy particles could not effectively adsorb onto the surfaces of the droplets as the ζ-potential value of its primary W_1_/O emulsion (PU-0) was detected to be −10 ± 0.2 mV (Figure 1d). However, the ζ potential of the W_1_/O/W_2_ emulsion droplets containing HIU-treated W_1_/O emulsions became progressively more negative. As a result, the droplets in these emulsions could be rationally coated by the soy protein particles.

#### 3.2.2 Double Emulsion Microstructure

Figure 1 (top right) shows CLSM images of double emulsions including HIU-treated W_1_/O (except DE-PU8) exhibiting the expected structures for W_1_/O/W_2_ emulsions with small water droplets trapped inside the larger oil droplets that were dispersed in water. Compared to the DE-PU0 and DE-PU2, the sizes of the O/W droplets in the DE-PU4 and DE-PU6 emulsions were smaller. The droplets of DE-PU4 and DE-PU6 emulsions were also homogeneously distributed throughout the continuous phase, and their internal structures were maintained (CLSM image of DE-PU8 was not provided). Microscopic images indicated that most of the aqueous phase droplets regarding DE-PU4 and DE-PU6 emulsions were submicron in diameter and uniformly dispersed into the oil phase. According to the PSD, the large droplets comprise over 90% of the total W_1_/O volume with the smaller submicron droplets representing less than 10%. Based on the microscopic images, the larger droplets appear most likely to be aggregates of the submicron emulsified droplets, formed during the emulsification process by collisions that occur simultaneously with size reduction in the presence of strong shearing forces. It was concluded that soy protein particles could produce a smaller droplet in W_1_/O/W_2_ emulsions with superior physical stability against coalescence because of irreversible particle adsorption at the O/W interface.^66^

#### 3.2.3 Flow Behavior and Viscoelasticity

**Figure 3:**
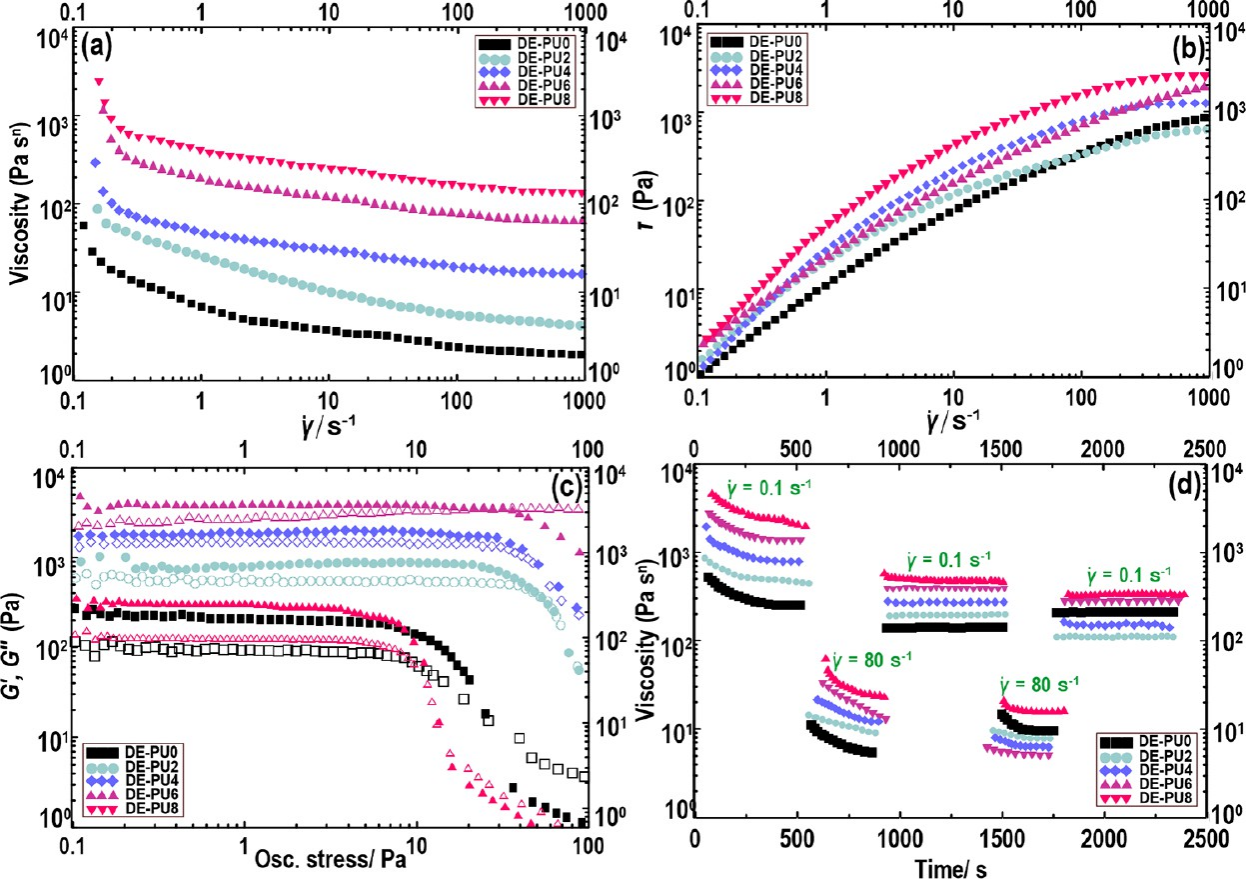
(a) Viscosity-shear rate curves; (b) shear stress-shear rate of double emulsions; (c) oscillatory stress sweep plots, where *G′* is solid symbols and *G″* is open symbols; and (d) 5-ITT of double emulsion variants.

To gain further insight into the suitability of double emulsions for the 3D printing process, the rheological properties of W_1_/O/W_2_ emulsions were assessed through flow, oscillatory, and thixotropic experiments (Figure 3). The shear rate dependency of the apparent viscosity (Figure 3a) or stress (Figure 3b) of soy-based double emulsions shows typical non-Newtonian behavior. As the apparent viscosity of W_1_/O/W_2_ emulsions reduced with increasing shear rate, the flow properties of all double emulsions, irrespective of the type of their formulations, were observed to exhibit a shear-thinning property (*n* < 1) (data not shown). The shear-thinning property of the double emulsions may be because of disruption and deformation of the flocs as the shear rate increases. Figure 3 also shows the maximum viscosity related to the double emulsions of DE-PU4 and DE-PU6, in which the apparent viscosity of the DE-PU6 emulsion reached approximately 500 Pa s. This is likely associated with a reduced droplet size (Figure 1) and formation of stronger aggregates (Figure 2).^48^ Moreover, the existence of soy protein particles promoted bridging flocculation of oil droplets (Figure 2). This can increase the effective volume fraction of the dispersed phase, if the aggregates have open, ramified structures (i.e., are fractal aggregates). Compared to the nonflocculated emulsion, both enhanced shear-thinning features and improved viscosity can occur in the flocculated emulsions as the aggregates preserve some of the continuous phases inside their structures, leading to increased viscosity.^66^ However, the viscosity of the DE-PU8 emulsion was decreased compared to DE-PU4 and DE-PU6, which could be due to an increased droplet size of this emulsion (Figure 1).

The oscillatory amplitude sweep experiment (Figure 3c) shows a noticeably greater storage modulus, *G*′ (τ), at lower amplitudes compared to the viscous modulus, *G*″ (τ). This obviously specifies an elastic gel property regarding all double emulsions, which follows the preceding flow property results (Figure 3a,b). Moreover, the moduli indicated that the double emulsion possesses a wider (>100 Pa) conventional LVR compared to those of high internal phase emulsion or emulsion gels.^66^ The obtained outcome demonstrates that the oil droplets may be close together through strong forces (e.g., intermolecular hydrogen linkages, bridging effect, and van der Waals forces), overwhelming high utilization stress, and are thus less susceptible to destruction. As can be observed from Figure 3c, the DE-PU6 and DE-PU4 emulsions presented a higher elastic gel (*G*′ (τ) > 10^3^ Pa) with a longer LVR (i.e., higher crossover points). This highlights that these double emulsions had a linear viscoelastic solid-like behavior (predominantly elastic) with high stiffness under the stress sweep (improved gel–sol transformation). The amplitude sweep data also provides evidence that DE-PU6 and DE-PU4 emulsions can reasonably increase the resistance of the system to any deformation. This phenomenon may be due to the soy protein particles promoting bridging flocculation in the system, which theoretically happens when a single particle attaches to a surface of more than one droplet. Typically, bridging flocculation (commonly in intermediate concentrations) includes a strong attractive interaction, which might be responsible for the stiffness of DE-PU6 and DE-PU4 emulsions.

The thixotropic properties of double emulsions were further evaluated to assess the relevant structure-recovery behavior of double emulsions through a 5-ITT (Figure 3d). It was detected that with increasing time in the first phase (at a low shear of 0.1 s^–1^) the viscosity was slightly reduced; in the meantime, at a high-shear of 80 s^–1^ the sensitivity became evident. However, the results for double emulsions revealed a high degree of recovery, even after five cycles alternating between 80 and 0.1 s^–1^. The suitability of double emulsions is likely considered for processing with a 3D extrusion printing system, in which a reforming network with reversible structure is extremely appreciated.^50^ Note that regarding the DE-PU6 emulsion a higher viscosity value was detected compared to that of other double emulsions. The difference in thixotropic properties could be attributed to the affecting the elastic and the viscous components of viscoelastic properties as a result of particle size change and the development of a flocculated system (Figure 2). This leads to different structural deformation properties.

#### 3.2.4 Nonlinear LAOS Rheology

**Figure 4:**
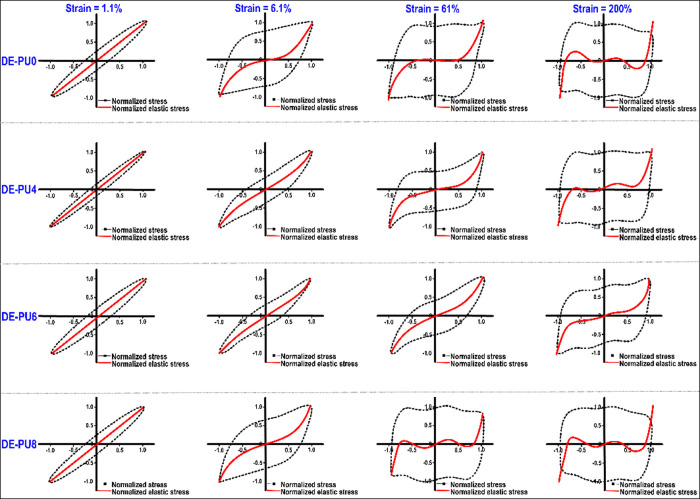
Area is surrounded by the “elastic” Lissajous-Bowditch plots as a function of amplitude in different double emulsions (a frequency of 1 rad s^–1^). Stress and strain results are normalized with a maximum stress/strain in the oscillation cycle.

The nonlinear stress response (i.e., strains beyond the LVR) can be detected by the LAOS experiment. LAOS offers a visual difference in the complex emulsion microstructure, which cannot be evaluated through a classical rheological experiment.^65^ Generally, the Lissajous-Bowditch curves obtained by LAOS can illustrate a quick assessment of the structural evolution in a real physical process like a 3D printing process, which accounts for the microstructure collapse of the system under large deformations.^65^ The instant intracycle stress of the double emulsions as a function of shear rate (applied strain) can detect viscous and elastic Lissajous-Bowditch plots. The viscoelastic moduli are independent of deformation rates throughout the LVR area, and the Lissajous curve is elliptical due to the sinusoidal oscillatory stress response. Following this area, elastic and viscous moduli mainly relate to the applied strain in the nonlinear region, where the presence of greater harmonics in the stress response leads to a twisted, nonsinusoidal shear stress waveform. Figure 4 shows the elastic Lissajous-Bowditch plots for double emulsions, assessed at dissimilar strains (1.1, 6.1, 61, and 200%) with a fixed frequency (1 rad s^–1^). The results of intracycle stress are normalized with respect to the maximum stress of the oscillation cycle. As can be seen, the deformation strain and the emulsion type strongly affect the shape of the Lissajous plots, where all of the double emulsions presented a perfectly elliptical shape at a strain of 1.1% (the curve of DE-PU2 not shown). This shows a mechanically stable viscoelastic double emulsion within the LVR, which is in accordance with the previous data for oscillatory amplitude sweep measurements (Figure 3c). The nonlinearity was detected as a distortion from an elliptical shape as the strain was increased (particularly for DE-PU0 and DE-PU8 emulsions), in which the level of deviation increased with increasing strain. This data show that DE-PU4 and DE-PU6 emulsions had more viscoelastic solid-like behavior (predominantly elastic) with high stiffness compared to DE-PU0, DE-PU2 (data not shown), and DE-PU8 samples. According to the literature, this particular distortion shape regarding Lissajous plots can be associated with dissimilar structure responses and microstructural properties of emulsions upon LAOS.^67^ The broader elliptical Lissajous plots (a strain of 1.1–6.1%) and an alteration to the parallelogram-like shape of the graphs (a strain of 61–200%) show an ultimate change from elastic- to viscous-prevailed properties, highlighting an increased viscous dissipation upon intracycle deformation, as well as highly non-linear mechanical response.

In the comparison of the Lissajous plots regarding double emulsion variants, the DE-PU4 and DE-PU6 inks (particularly DE-PU6) presented inferior distortion from their initial elliptical geometry and normally minor surrounded region of the circles with increasing strain (especially at a strain of 1.1, 6.1, and 61%) compared to the rest of double emulsions. This means that the microstructures of DE-PU0, DE-PU2 (data not shown), and DE-PU8 emulsions were less elastic with lower durability compared to DE-PU4 and DE-PU6 samples. Therefore, they are more likely to fracture upon large deformations (like processing during 3D printing), which results in a more pronounced degree of nonlinear response. Besides, a constant upturn of the decomposed elastic stress was detected in the DE-PU4 and DE-PU6 emulsions within the strain range between 1.1 and 6.1%. This signifies that these samples hold more of their elastic character and even show a slight number of intracycle strain rigidifying.^65^ Contrary, the DE-PU0, DE-PU2, and DE-PU8 emulsions already show yielding at the strain of 6.1%. As aforementioned, the DE-PU4 and DE-PU6 emulsions presented a more reduced droplet size containing droplet-rich domains (Figure 1e,f), an increased viscosity (Figure 3a), and more thixotropic behavior (Figure 3d). It is worth noting the yielding of DE-PU6 emulsion happened at a strain above 61% strain, which suggests an evolution from the elastically rigidifying compound to the shear-thinning fluid-like compound (the strains of 61–200%). It should be kept in mind that though the DE-PU4 and DE-PU6 emulsions showed somewhat comparable linear viscoelastic features within the LVR (Figure 3c), they presented considerably dissimilar nonlinear viscoelastic responses upon LAOS measurements. This highlights more valuable evidence concerning structural alterations under large deformation and applied applications and processing (like 3D printing) of these emulsion systems.

**Figure 5:**
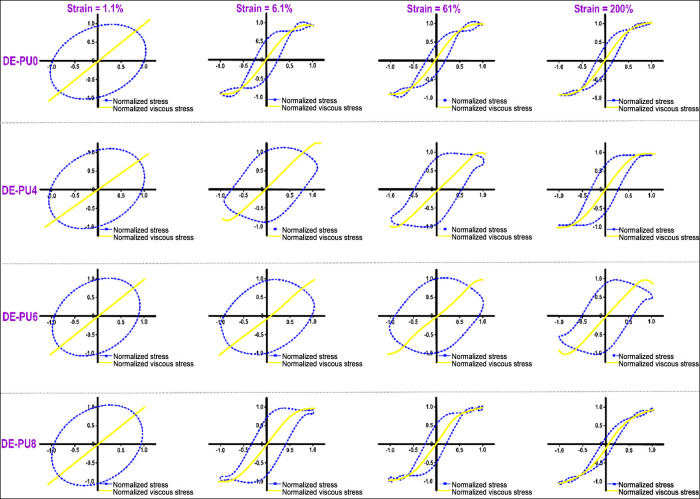
Area is surrounded by the “elastic” Lissajous-Bowditch plots as a function of amplitude in different double emulsions (a frequency of 1 rad s^–1^). Stress and strain results are normalized with the maximum stress/strain in the oscillation cycle.

Figure 5 shows the normalized viscous Lissajous plots of different double emulsions, which provides valuable evidence of structural evolutions upon large deformation. A strong nonlinear viscoelastic property with high shear-thinning at higher strain rates can be observed when the shear stress–strain rate loops are gradually changed from the elliptical to a near S-shape.^65^ With increasing strain from 1.1 to 61%, DE-PU4 and DE-PU6 emulsion-based inks (especially DE-PU6) showed only a minor distortion from their initial shape, where the change of the surrounding zone of the loops was not as apparent. Regarding DE-PU0, DE-PU2 (data not shown), and DE-PU8 samples, it is found that the nonlinear viscous contribution appeared at a strain of 6.1%, as demonstrated by the rhomboidal form of the Lissajous plots with a slope change of the decomposed stress plots (Figure 5). Once again, this indicates that DE-PU4 and DE-PU6 inks presented a more elastic solid-like feature, in accordance with the aforementioned measurements of the elastic Lissajous plots (Figure 4). It should be noted a secondary loop was detected in the stress–strain rate plots with additional increasing strains (61–200%), particularly regarding DE-PU0 and DE-PU8 inks. The obtained property relates to a high level of nonlinearity in the elastic stress, where a secondary loop characteristically emerges once the time scale for the microstructure reorganizations is shorter compared to that of the deformation.^65^ Moreover, it is most probably accountable for the stress overshoots noticeable regarding the obtained elastic contribution to the stress just following yielding in the elastic Lissajous plots (Figure 4).

Shear recovery experiments were performed to evaluate the rheology time dependence of the inks after printing. To correlate these measurements with the actual printing shear rate, the maximum shear rate (MSR) was evaluated. The shear rate in a 3D printer nozzle for Newtonian behavior is defined as
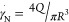
2Where *R* is defined as the radius of the pipe, and *Q* is the volumetric flow rate. For shear-thinning fluids (pseudoplastic), Rabinowitsch correction is necessary. For the power-law model (η = *k*γ̇ ^*n*–1^) the shear rate
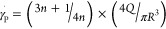
3

Not surprisingly, progressing to lower frequencies but higher strain amplitudes (i.e., increasing the MSR) leads to a better model agreement, as the stress response is dominated by the viscous behavior of a relatively unstructured material. Depending on the calculation, the MSR of the printing system was detected to be between 60 and 423 s^–1^ with the consideration of the rheology dependence on the emulsion type. Reducing the frequency and strain amplitude each by an order of magnitude locates the LAOS conditions at sufficiently small strain rates and strains such that the experiment is largely dominated by the elasticity leading to the yield stress (Supporting Information, Section S7). Note that this strain value is still above zero deformation strain such that the dispersion is in the weakly nonlinear regime. The experimental data show predominantly elastic behavior, with a stress overshoot and viscous flow evident as the maximum shear rates are reached.

### 3.3 Characterization of 3D-Printed Constructs

#### 3.3.1 Printing Quality

**Figure 6:**
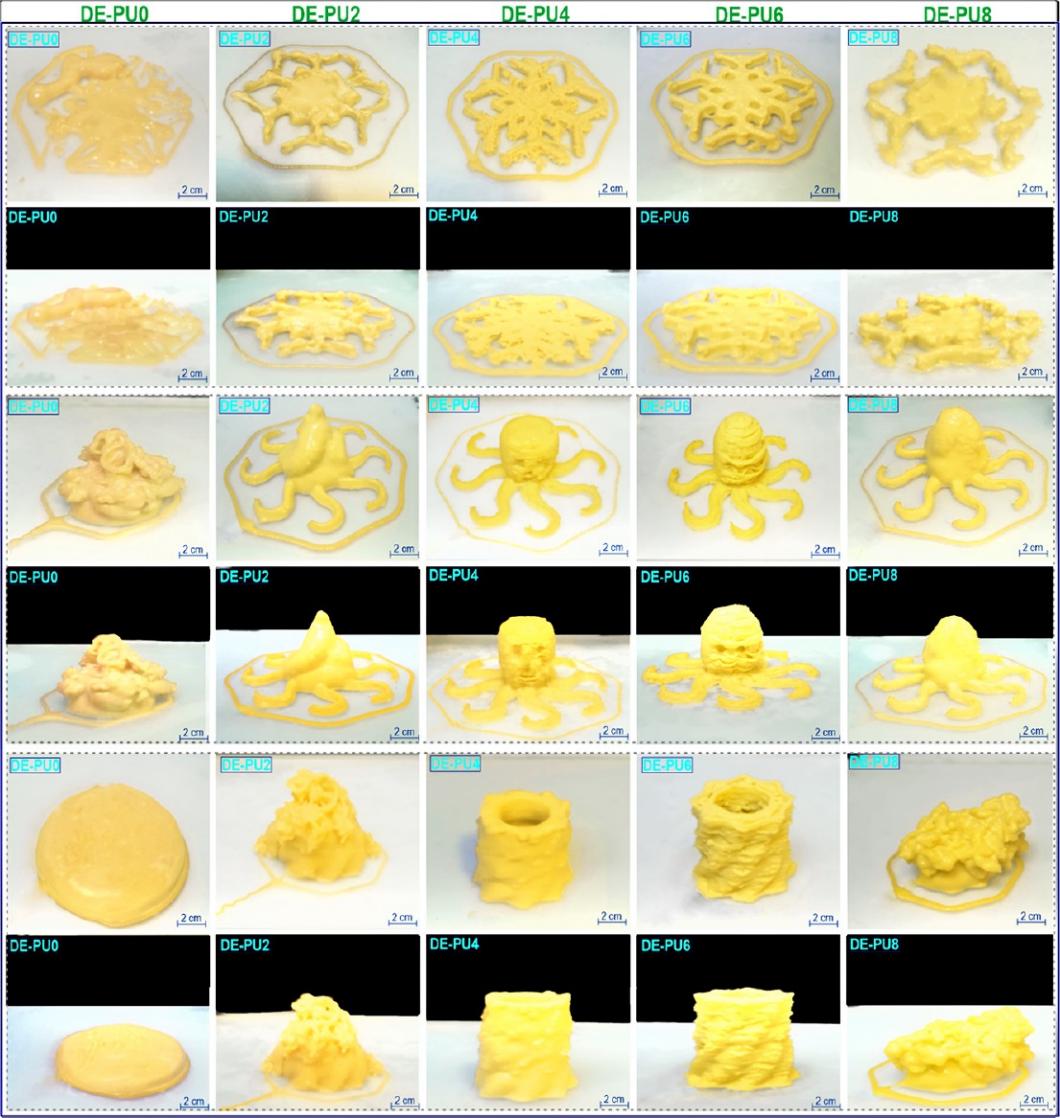
Printing performance of the double emulsions into different 3D-printed objects.

One of the main intentions of this study was engineering printing inks to be effectively processed with an extrusion-based printer, manufacturing diverse shapes of 3D-printed objects with higher shape fidelity, enhanced printing precision, and good resolution of the deposited layers. To evaluate the 3D printing performance for different double emulsion-based inks, the prepared double emulsion-based inks were printed using a layer-by-layer deposition to construct different 3D shapes of the snowflake, octopus, and cylindrical ones (Figure 6). The printing performance shows the quantitative basis to evaluate and quality control of 3D-printed architectures, offering the integrity of the printing geometry and the clarity of the printed layers.^34^ All double emulsions could be successfully extruded during printing, although DE-PU0 ink displayed a sagging structure, printing layer fusion, and phase separation (Figure 6). Compared to DE-PU0, though DE-PU2 ink showed a somewhat better-printed layer following 3D printing, its 3D structures were still uneven with higher defects, which had a higher susceptibility to collapse and cracking. Interestingly, the printed layers of cylindrical shape regarding both DE-PU0 and DE-PU2 inks collapsed and merged with each other (due to the higher height of the printed model); thus, the resulting shapes could not be effectually printed. Obviously, the snowflake, cylindrical, and octopus printed from DE-PU4 and especially DE-PU6 inks revealed outstanding layer resolution and shaped fidelity. In conjunction with the flow behavior (Figure 3a), thixotropic properties (Figure 3d), and nonlinear viscoelastic features (Figure 4) of double emulsions, it was mainly concluded that the prepared DE-PU4 or DE-PU6 inks showed outstanding viscoelasticity, improved thixotropic recovery, and larger nonlinear elastic characters, which made these inks perform well in 3D printing. Consequently, the double emulsion inks could develop functional bioactive 3D-printed shapes with diverse structures. On the other hand, ink 3D-printed objects resulting from DE-PU8 did not maintain their shape precision and structural integrity compared to DE-PU4 or DE-PU6 inks (Figure 6). A conclusion for unsuccessful printed shapes of DE-PU8 printed structures may be associated with its weak gel network, unstable colloidal system, and less recoverable features.

#### 3.3.2 Microstructure of 3D-Printed Structures

**Figure 7:**
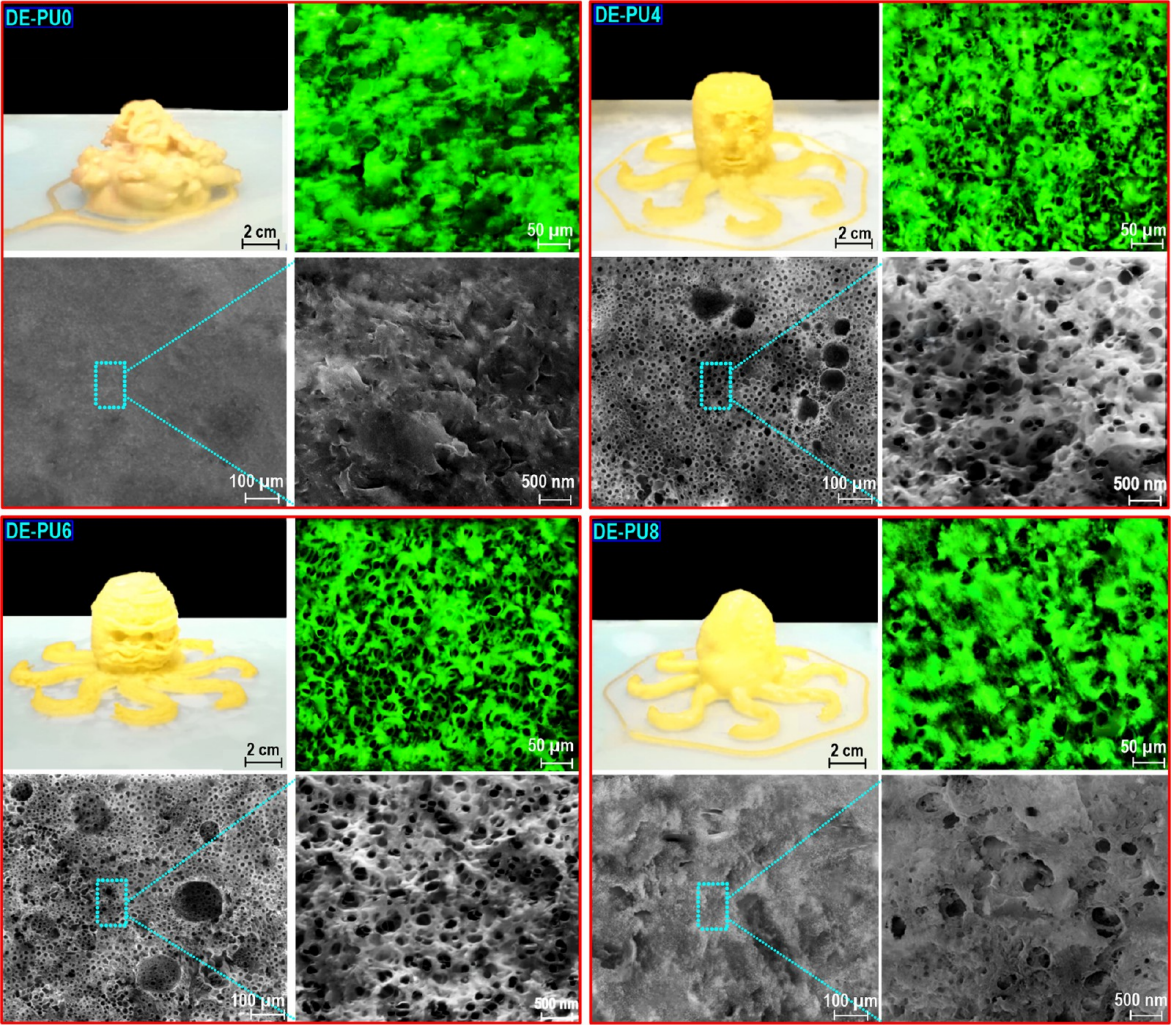
Microstructure comparison of different printing objects with regard to the fluorescence images (50 μm) and FE-SEM images at different magnifications (100 μm and 500 nm).

An FE-SEM evaluation was accomplished on the surface of the 3D structure to evaluate the effect of different types of printable double emulsion inks on the morphology and microstructures of printed architectures (Figure 7). Clearly seen in the FE-SEM image, the microstructures of the 3D-printed DE-PU0 structure were characterized by an unambiguous compact wall with a high level of irregularity and no apparent pore structure inside its matrix. Similarly, the surface morphology of printed DE-PU2 seemed also to be rugged with an irregular microstructure (image not shown). Interestingly, printed DE-PU4 and DE-PU6 showed a 3D interconnected porous structure and blurry sieve-like ones having several aperture diameters in nanometers size. Compared to DE-PU4, DE-PU6 showed a more randomly opened macroporous structure in the 3D interconnected framework with a more uniform and regular pore structure having a pore size varying from 100 to 500 nm. This designates a slightly denser matrix, leading to a better structural and mechanical stability. A macroporous 3D structure affects the improved printing quality and dimensional stability of the printing architectures.^37,50^ These findings concern those for a higher recoverable structure, strong gel-like network, and more viscoelastic solid-like behavior of doubles emulsions. Distinguished from the interconnected macroporous structure of DE-PU4 and DE-PU6, much less riddled holes with diameters around 10–500 nm were observed in the microstructure of the DE-PU8. The resulting firmer pore walls in DE-PU4 and DE-PU6 offered a more supporting capacity and therefore may clarify a more suitable compressibility, which may have potential applications as bioscaffolds, cell culture, substrates for drug delivery, catalyst carriers, etc.

The fluorescence images of 3D-printed objects also showed that the DE-PU4 and DE-PU6 offer a macroporous structure (Figure 7). It is obvious that these 3D structures had fairly larger pore sizes compared to the droplet sizes of the double emulsion-based inks (Figure 1). Upon the preparation of 3D structures, a drying process was achieved to enhance the porosity and interconnectivity of the 3D structures. During this drying process, the size of the oil droplet would be increased. Thus, the pore sizes of as-prepared 3D-printed structures were larger compared to the diameters of related emulsion-based inks. It has been reported that the pore size of 3D structures is relatively associated with the droplet sizes of the precursor emulsion-based inks.^10^ Consequently, it led to the conclusion that the integration of the double emulsion templating technique into 3D printing successfully resulted in engineering a 3D structure with a controllable porous matrix.

**Figure 8:**
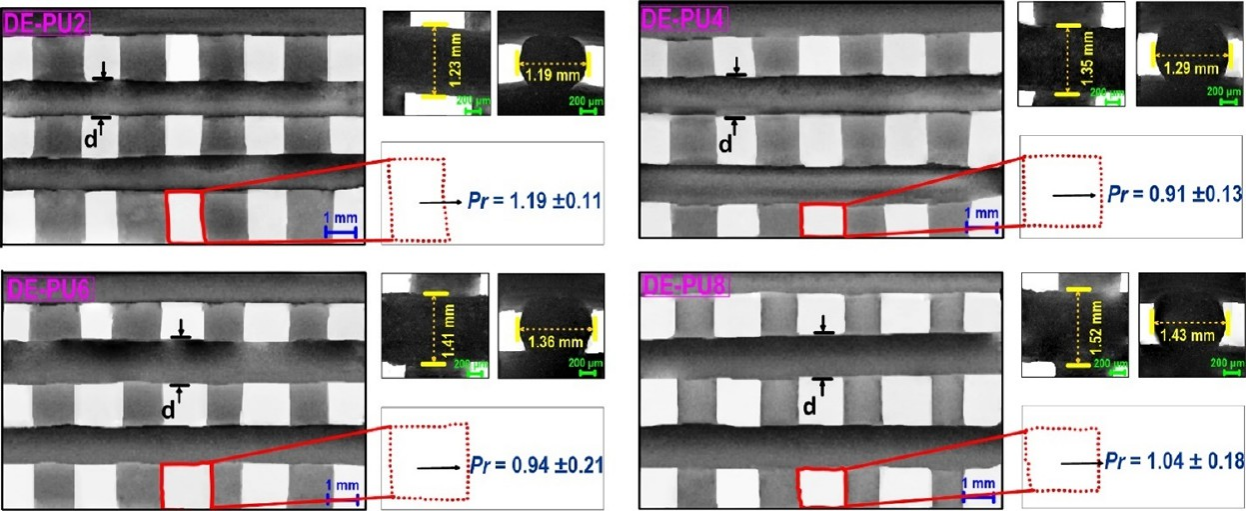
SEM images of 3D-printed grids as well as their relevant photomicrographs of the surface or cross-sectional rupture of different printed filaments.

The printed pattern precision of an axial pore in an *XY* plane to exhibit a perfect square geometry was related to *Pr* (0 ≤ *Pr* ≤ 1). In a particular shape, a square geometry (high precision) can be measured if *Pr* = 1, an irregular shape if *Pr* > 1, and a round shape if *Pr* < 1. As Figure 8 shows, the *Pr* was detected to be 1.13 ± 0.11 for DE-PU0 (not provided) and DE-PU2, whereas the *Pr* was found to be 0.91 ± 0.05 and 0.94 ± 0.04 for DE-PU4 and DE-PU6, respectively, showing better printing performance. In addition, the pattern geometry of the latter printed objects was somewhat rounded, as the *Pr* values were close to 1. It is concluded that the shape fidelity of DE-PU4 and DE-PU6 was upheld even if more layers were added to the structures.

#### 3.3.3 Mechanical Strength of 3D-Printed Constructs

**Figure 9:**
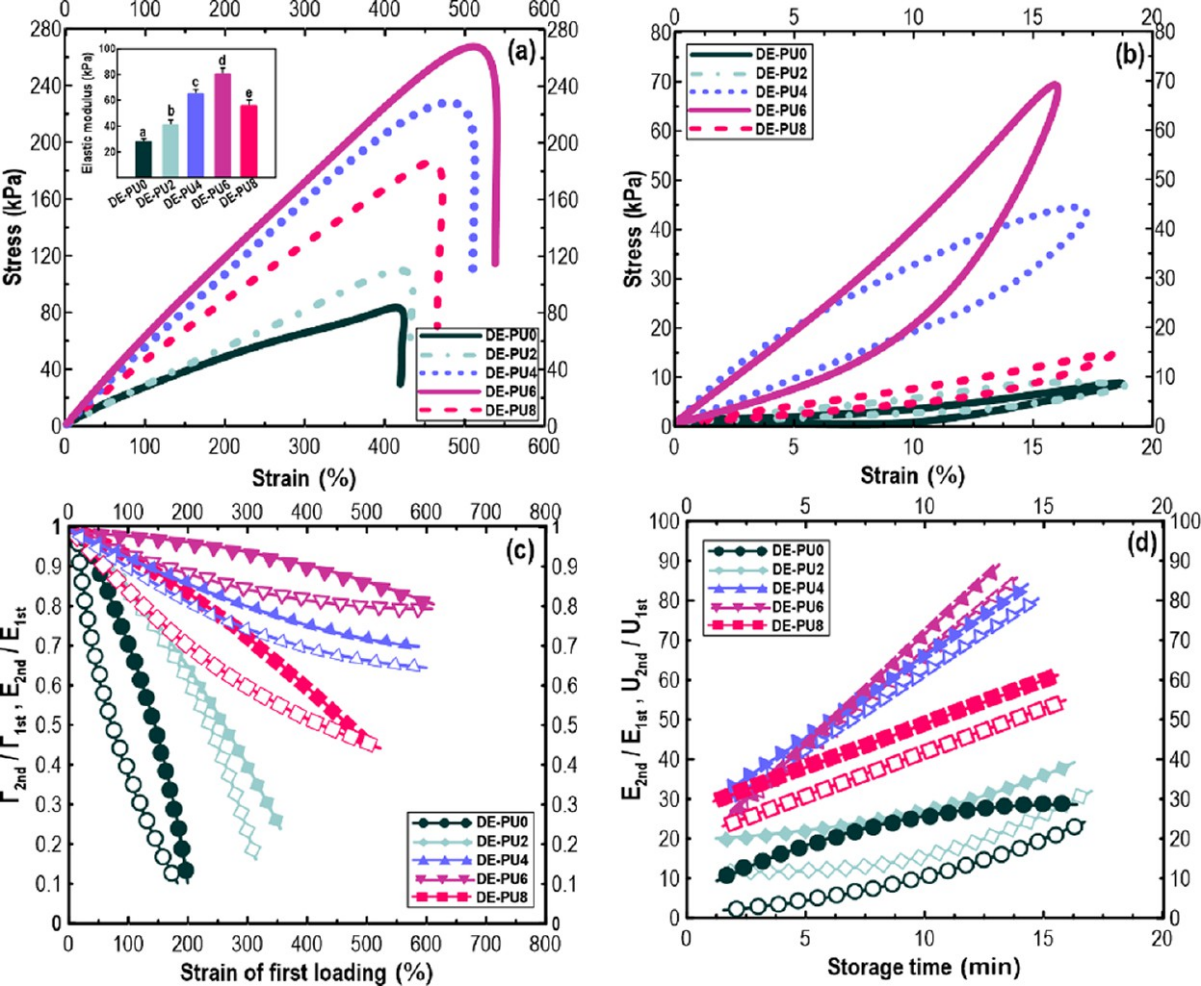
(a) Stress–strain curves of different 3D printing architectures. (b) Curves of loading–unloading cycles. (c) *Γ*_second_ /*Γ*_first_ and *E*_second_ /*E*_first_ as a function of the strain of first loading in different printed objects. (d) Proportion of elastic modulus and energy dissipation (*U*) upon the second loading–unloading cycle to those during the first one for the relaxed and notched samples kept at 90 °C was plotted against different storing times.

The mechanical properties (including elastic modulus (*E*), fracture energy (*Γ*), and toughening mechanism) of the 3D-printed objects were measured. The mechanical data showed that the lowest elastic modulus, *′E′* was detected to be 32 and 32 kPa for DE-PU0 and DE-PU2, respectively, whereas its value was maximum for DE-PU4 and DE-PU6 with a value of 32 and 32 kPa, respectively (Figure 9a). As discussed in Sections 3.2.3 and 3.2.4, the DE-PU4 and DE-PU6 emulsion-based inks presented a higher elastic gel with a highly mechanically stable viscoelastic character. This highlights that these double emulsions had a linear viscoelastic solid-like behavior (predominantly elastic) with high stiffness under the stress sweep (improved gel–sol transformation). Compared to DE-PU4, 3D-printed DE-PU6 offered a greater toughness, in which the maximum fracture energy was attained.

The toughening phenomenon and fracture mechanism of printed structures were further assessed by using the loading/unloading evaluations. As shown in Figure 9b, below the yield strain of printed samples (at a small tensile strain), DE-PU6 had an obvious hysteresis feature and preserved a considerable level of enduring deformation upon unloading. Likewise, DE-PU4 exhibited a typical degree of hysteresis, although other printed structures did not show a hysteresis behavior.

To measure a typical disrupting strength, a ratio of fracture energy, *Γ*_second_ (or elastic modulus, *E*_second_) in the second loading–unloading phase compared with their values in the first loading–unloading phase (*Γ*_first_ or *E*_first_) was considered. As shown in Figure 9c, all samples exhibited an obvious decrease of the *E*_second_/*E*_first_ or *Γ*_second_/*Γ*_first_ with increasing strain in the first loading–unloading cycle. This indicates that the elastically printed matrices were broken with an increase in the extension level. According to the previous data on the greatest *'E'* values of DE-PU4 and DE-PU6, this result could be attributed to the higher viscoelasticity, greater viscosity, and superior thixotropic properties of the relevant inks. On the other hand, Figure 9d shows the recoverability of the notched 3D printing structures. For both PU4 and DE-PU6, the *'E'* parameter and energy dissipation (*U*) were found to be recovered to around 80 and 65%, respectively, presenting a brilliantly recoverable structure. The disrupting strength results well agree with the 5-ITT (Section 3.2.3). It has been reported that the energy dissipation in a multisystem emulsion system is positively related to a decreased droplet size having bridging flocculation of the oil droplets, in which such dissipated energy may be recovered after relaxation.

#### 3.3.4 Tribology

**Figure 10:**
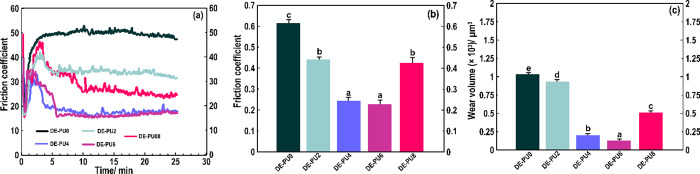
(a) Friction coefficient curves. (b) Average friction coefficient and (c) wear volume.

Figure 10a–c shows the friction coefficient curves and average friction coefficients and wear volumes in different printed samples. Compared to the DE-PU0 and DE-PU2, lower friction coefficients were detected for DE-PU4, DE-PU6, and DE-PU8. Specifically, the friction coefficient initially attained the highest value at around 2 min, and after that started to decay. It was assumed that the turning point was associated with a time once the 3D structures with satisfactory mechanical strength developed. In the meantime, the friction coefficient plot tended to be more stable and smoother regarding printed DE-PU4 and DE-PU6. The friction coefficient concerning DE-PU6 was 0.212, which decreased by about 75% compared to that of DE-PU0 (0.602). Similarly, the mean wear volume (Figure 10c) for DE-PU6 was also reduced by 75% compared to that of DE-PU0. This is likely associated with the improved antiwear and self-lubricating performances of these types of printed double emulsions.^66^

#### 3.3.5 Cell Encapsulation and Cell-Loading Bioprinting

**Figure 11:**
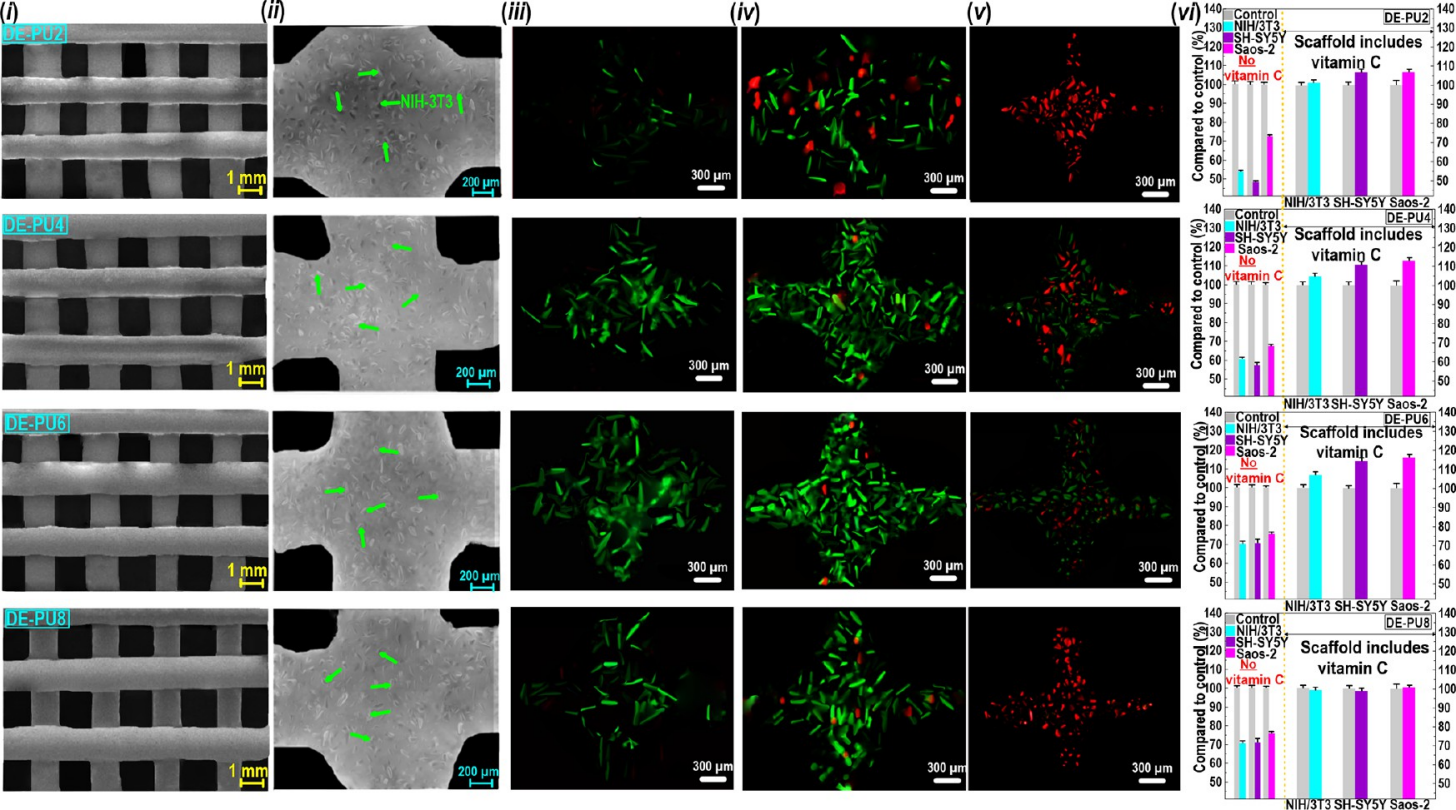
(i) Representative images of a one-layer printed grid immediately after printing. (ii) Cell viability of 3D-printed scaffolds immediately after printing at a cell density of 1× 10^7^ cells mL^–1^ (green arrows represent NIH/3T3 cells). Live/dead assay of NIH/3T3 cells encapsulated into the printed one-layer grid of scaffolds (containing encapsulated vitamin C) immediately after printing (iii) and 1-week cell culture (iv). (v) Live/dead assays of NIH/3T3 cells encapsulated into the printed one-layer grid of scaffolds with no encapsulated vitamin C. (vi) Cell viability of NIH/3T3 (cyan bars), Saos-2 (purple bars), and SH-SY5Y (magenta bars) in comparison with control conditions (gray bars).

Because of mimicking the imperative multifunctionalities of ECM, the gel-like double emulsions show a promising material for cell culture.^53−56^ This type of emulsion can produce a swollen matrix possessing outstanding mechanical properties,^52,56^ cell adhesion, and antiwearing features^57,58^ similar to soft tissues. Moreover, due to the easy modification of gel-like double emulsion compositions, they reveal effective biological functionality, which endows a suitable environment for cell proliferation.^59,60^ The effective gel-like double emulsions synthesized in the current study suggested a mechanical strength with the range of elastic modulus of soft tissues and enhanced thixotropic features with a recoverable matrix and self-lubricating property.^65^ These functionalities are likely to contribute to the outstanding biological response and regeneration of complex living tissues. To assess the capability of our fabricated 3D-printed scaffolds to encapsulate cells and to monitor cytotoxic degradation products and biocompatibility, we performed the survival of cells in different biological environments after encapsulation for up to 1 week (columns (i*–*vi) in Figure 11). An indirect assay was conducted to verify the biocompatibility of the developed 3D-printed scaffolds (Supporting Information, Section S8). To evaluate the feasibility of this type of scaffold in diverse biological environments, three different cell lines were utilized (column (vi) in Figure 11; tables for cell viability). As can be seen, there was no considerable change in the viability of neuronal-like cells (SH-SY5Y), osteoblast-like cells (Saos-2), and fibroblasts (NIH/3T3) in the printed degradation products, which verified the biocompatibility of the 3D-printed scaffolds.^68,69^ Further, we evaluated the effect of vitamin C on cell viability through indirect tests. It was obvious that the 3D-printed scaffolds containing vitamin C offered more viable cells of SH-SY5Y, Saos-2, and NIH/3T3 compared to 3D-printed scaffolds with no encapsulated vitamin C.

NIH/3T3 cells were then considered to be 3D printed at a high cell seeding density (1 × 10^7^ cells mL^–1^) to measure cell viability during printing (Supporting Information, Section S8). The one-layer printed grids showed well-defined printed architectures with good printing resolution (column (i) in Figure 11). As expected, these printed structures revealed better printing quality than those illustrated in Section 3.3.1, which could be due to a lower number of deposited layers. Immediately after printing, the NIH/3T3 cells presented a somewhat rounded shape, signifying the adhesion process had not happened yet (column (ii) in Figure 11).^65^ In this case, the live/dead test of NIH/3T3 cells encapsulated into one-layer printed grids also showed a small fraction of cell density, especially for DE-PU2 and DE-PU8 (column (iii) in Figure 11). However, DE-PU4 and DE-PU6 showed moderately high cell viability. After prolonging the cell culture for up to 1 week, the cells spread within the bioinks, where the reticulate F-actin filament of NIH/3T3 cells was clearly observed (column (iv) in Figure 11). In contrast, DE-PU0 (not shown) and DE-PU2 propose relatively low cell viability with a large fraction of dead cells throughout the culture period. Compared to scaffolds containing vitamin C, printed scaffolds with no encapsulated vitamin C did not show high cell viability within the printed grid after 1 week of cell proliferation (column (v) in Figure 11). This emphasizes the positive impact of vitamin C on the proliferation of NIH/3T3 cells.^67^ It was reported that oxidative stress and the subsequent DNA damage can be avoided through the activity of vitamin C.^68^ For example, Liao et al.^69^ demonstrated that vitamin C efficiently quenched singlet oxygen (^1^O_2_), subsequently decreasing oxidative damage resulting from chlorin e6 (Ce6)-mediated photodynamic therapy on NIH/3T3 cells. Our results also have verified the capability of the bioactive 3D-printed scaffolds containing vitamin C into a multifunctional biocompatible 3D structure, along with their potential to offer cell growth while preventing NIH/3T3 cells oxidative damage in complex printed architecture.^70^

## 4 Conclusions

In summary, vitamin C was encapsulated within an inner water phase of W_1_/O/W_2_ emulsions polyglycerol polyricinoleate as a hydrophobic emulsifier and soy protein particles as a hydrophilic emulsifier, which could be printed as a hierarchically microporous structure for the applicability of the developed bioink for biomimetic scaffolds. The gel-like double emulsions were further produced to offer cell encapsulation and cell-loading bioprinting. The emulsion properties (microstructure, static and dynamic rheology, particle size and distribution, and nonlinear rheology), printing quality, and tribology measurements of double emulsions, as influenced by HIU time, were evaluated. Phase separation and a bimodal distribution with a decreased nonlinear property under large amplitude oscillatory shear stress of double emulsions were largely reduced upon the application of power ultra sonication. The outstanding flow behavior broadens the potential of double emulsion in the development of 3D-printed porous structures, in which the 3D-printed double emulsion-based inks showed high shape fidelity and integrity. Further, a high level of porosity with a uniform structure in terms of orientation and shape of spaces was observed in the 3D-printed objects. The printed scaffolds with encapsulated vitamin C induced high cell viability within the printed grid after 1 week of cell proliferation. This emphasizes the positive impact of vitamin C on the proliferation of NIH/3T3 cells. These results indicate that vitamin C-loaded gel-like double emulsions enhanced the cellular affinity, cell biocompatibility, and dimensional stability of the 3D-printed scaffolds under the physiological conditions, which has great potential to be utilized in tissue engineering applications.

## Supporting Information Available

Dilution test; the appearance of the primary and secondary emulsions; effect of pH on colloidal stability of double emulsions; encapsulation efficiency of vitamin C in double emulsions; printing setup; microstructure comparison in the emulsion with and without soy protein particles; estimating printing maximum shear rate (MSR); and cell viability assay: indirect tests (PDF)
