# Supplementary material for: 3D Printing of Bioactive Gel-like Double Emulsion into a Biocompatible Hierarchical Macroporous Self-Lubricating Scaffold for 3D Cell Culture
Source: ACS Appl Mater Interfaces. 2023 Oct 12;15(42):49874–91. doi: 10.1021/acsami.3c12078 (PMC10614201; doi:10.1021/acsami.3c12078)
Supplement: Supplementary file 1 — am3c12078_si_001.pdf [file am3c12078_si_001.pdf]

SUPPORTING INFORMATION:

3D Printing of Bioactive Gel-like Double Emulsion into a Biocompatible Hierarchical Macroporous Self-lubricating Scaffold for 3D Cell Culture

Mahdiyar Shahbazi[a], Henry Jäger[a*], Adeleh Mohammadi[b], Peyman Asghartabar Kashi[c], Jianshe Chen[d], Rammile Ettelaie[e]*

[a]*Institute of Food Technology, University of Natural Resources and Life Sciences (BOKU), Muthgasse 18, 1190 Vienna, Austria*

[b]*Faculty of Food Science and Technology, Gorgan University of Agricultural Sciences and Natural Resources, Gorgan, 4913815739, Iran*

[c]*Faculty of Biosystem, College of Agricultural and Natural Resources, Tehran University, 31587-77871 Karaj, Iran*

[d]*Food Oral Processing Laboratory, School of Food Science & Biotechnology, Zhejiang Gongshang University, Hangzhou, 310018, China*

[e]*Food Colloids and Bioprocessing Group, School of Food Science and Nutrition, University of Leeds, Leeds, LS2 9JT, UK*

Corresponding Authors:

*Mahdiyar Shahbazi - Institute of Food Technology, University of Natural Resources and Life Sciences (BOKU), Muthgasse 18, 1190 Vienna, Austria; Orcid https://orcid.org/0000-0002-2485-9130; Email: mahdiyar.shahbazi@boku.ac.at; shahbazim00@yahoo.com.

*Henry Jäger - Institute of Food Technology, University of Natural Resources and Life Sciences (BOKU), Muthgasse 18, 1190 Vienna, Austria; Email: henry.jaeger@boku.ac.at

S1. Dilution test

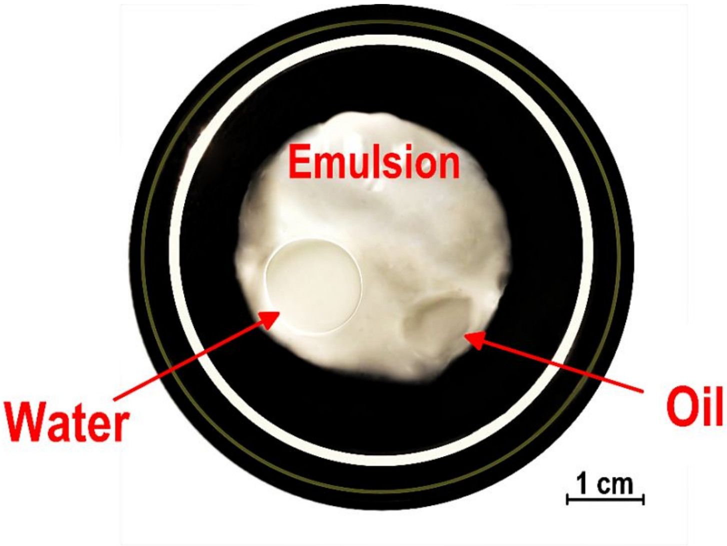

Figure S1. Continuous phase test of the emulsion by the method of dilution test.

S2. The appearance of the primary and secondary emulsions:

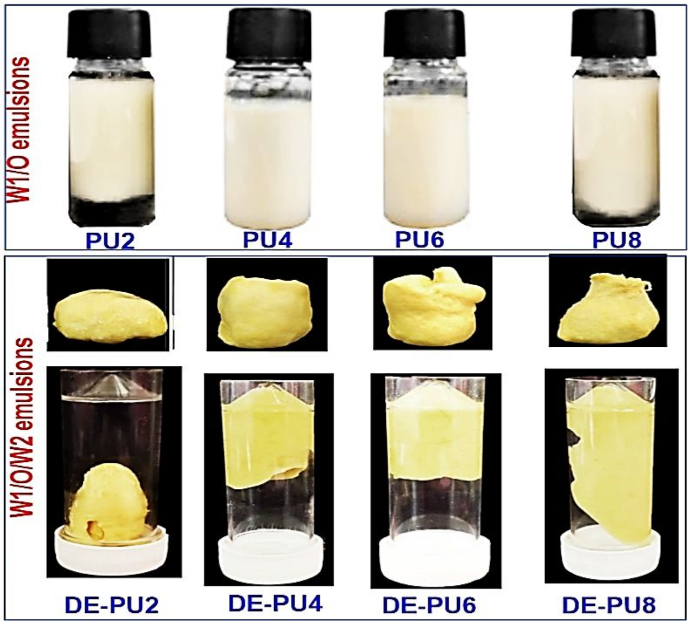

Figure S2. Visual observation of single emulsions and double emulsions (24 h after preparation).

S3. Effect of pH on colloidal stability of double emulsions

Further, in the current project, when the pH was decreased to <6, the $W_1/O/W_1$ emulsion (containing soy protein particles) showed a characteristic phase separation (Figure S3). As Figure S3 shows the double emulsion presented a typical phase separation when the dispersion pH was in the range of 3.5–6. This phenomenon might be resulted from the precipitation of soy protein near to its isoelectric point (pI of approximately 5), which would induce protein aggregation. Then, this colloidal-based dispersion including the precipitated soy particles with higher particle sizes could not effectively contribute to the Pickering stylization process. But these aggregates were redispersible when the dispersion pH was out of this range (<3 or > 6) (Figure S3), which the double emulsion was physically stable in these pH ranges. However due to the inhabitation of cell growth (presented in 3.3.5 in the revised manuscript), we didn't use the pH < 3.

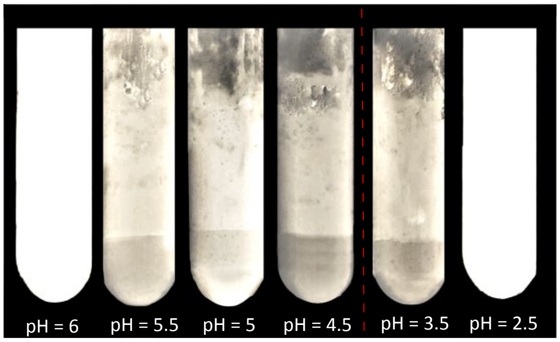

Figure S3. Visual observation of double emulsions in different pH values (48 h after preparation). (To better show a phase separation in double emulsions in different pH values, we used a Black & White mode)

S4. Encapsulation efficiency of vitamin C in double emulsions

Methods

The encapsulation efficiency of vitamin C was determined in fresh double emulsions and after storage (3, 24 and 48 h) at 40 °C. The double emulsions were diluted (1:1) in 0.001 M phosphate buffer (pH = 6.8) containing 0.1 M NaCl and centrifuged at 500 × *g* for 15 min at 23 °C. The subnatant was collected and centrifuged again at 7500 × *g* for 30 min at 23 °C. Proteins in the subnatant obtained after the second centrifugation were precipitated by addition of 20% trichloroacetic acid solution (1:1) and removed by centrifugation at 3000 × *g* for 15 min at 23 °C. The vitamin C concentration in the supernatant was determined by measuring the absorbance at 361 nm according to the method of O'Regan and Mulvihill (2009)

and by taking into account the dilution with trichloroacetic solution. Encapsulation efficiency [*E* (%)] of vitamin C in double emulsions was calculated using Eq. (S1):

$$\mathrm{E}\ (\%) = \left({C_{w1}X_{w1} - C_s\,(D + X_{w2})} \middle/ {C_{w1}X_{w1}} \right) \times 100 \qquad \text{(S1)}$$

where C_{W1} is the initial vitamin C concentration in the internal aqueous phase of the emulsion (0.2%) and C_S, the vitamin C concentration in the subphase collected after centrifugation of diluted emulsion; X_{W1} and X_{W2} are, respectively, the mass fractions of the internal (0.07) and external (0.65) aqueous phase of the emulsion and *D* is the dilution volume (*D* = buffer volume/emulsion volume).

Results and discussion

A coalescence index can be calculated from the rate of change in droplet diameter over the 48-h storage period (Figure S4). Compared with non-sonicated sample (PU-0), HIU sonication notably decreased the coalescence index of the emulsions of PU-2, PU-4, and PU-6. Higher sonication time was shown to reduce the initial droplet size (Figure 1 in the main body of paper) and could also increase the packing of surfactant molecules at the interface. These two factors have been shown to reduce the coalescence rate of emulsion droplets (McClements, 2005). An additional time of 8 min in the sonication (PU-8) had no significant effect on the coalescence index ($p > 0.05$). Although 8 min-sonication reduced the initial droplet size (Figure 1 in the main body of paper), this effect was associated with the disruption of droplets aggregates. An additional time of 8 min is unlikely to affect the effective droplet size or the packing of surfactant molecules at the interface.

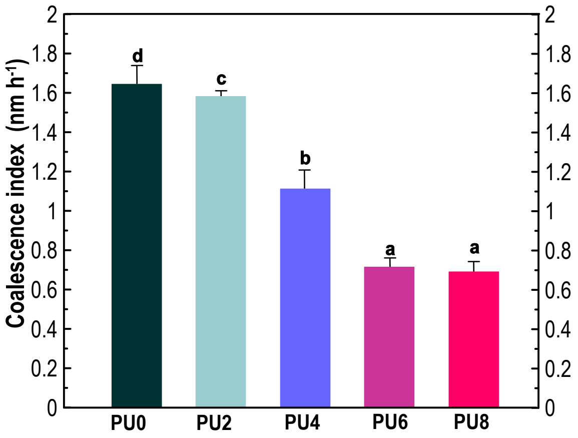

Figure S4. Coalescence index (CI) of W_1/O primary emulsion droplets for different samples. *CI* was calculated from the increase of droplet diameter (*d*) over the 48-h storage period (*t*). CI = Δ*d*/Δ*t*. Error bars represent the standard errors obtained from the statistical model.

The stability of primary emulsions was monitored during storage using a vertical optical scan analyzer; no signs of phase separation were observed (data not shown). Considering the average droplet size (Figure 1 in the main body of paper), the viscosity of oil and the density difference between the two phases, Stokes law predicts a sedimentation rate of 0.5–2 μm h^{-1}, which will not give a detectable phase separation after 48 h storage.

The vitamin C concentration in the external aqueous phase was measured to calculate the encapsulation efficiency. An encapsulation efficiency higher than 90% was obtained for the sonicated emulsions, indicating that the vitamin C remained entrapped within the internal aqueous phase. Storage time and external aqueous phase had no significant effect ($p > 0.05$) on encapsulation efficiency (data not shown). However, efficiency was significantly affected ($p \leq 0.05$) by the emulsification conditions (Table S1). The difference is likely associated with aggregation/coalescence of water phase droplets inside the primarily developed W_1/O emulsion. Encapsulation efficiency was higher (98.4%) in the case of emulsification with 4- and 6-min sonication. The sonication produced small droplets (Figure 1 in the main body of paper) with a smaller interfacial area, which minimized close contact between the internal and external aqueous phases. These emulsions showed lower phase separation than double emulsions produced by 2- and 8-min sonication (Figure S4).

Table S1. Effect of emulsification conditions on encapsulation efficiency of vitamin C in $W_1/O/W_2$ double emulsions.

Emulsification conditions	Encapsulation efficiency (%)
PU-0	17.4^{a}
PU-2	90.1^{b}
PU-4	94.4^{c}
PU-6	96.2^{d}
PU-8	91.4^{b}

[a]Values given have a standard error of 0.3; values with different superscript letters within the column are significantly different at $p \leq 0.05$.

S5. Printing setup

Prusa i3 is named after the third repetition of the design by Josef Průša. All parts of this 3D printing system were open-source and were part of the 3D Soft-Gel Printer project. Table S2 resumes the main characteristics depicts the original Prusa i3 Printer assembly kit and an assembled printer.

Table S2. Technical specifications of Prusa i3.

Technical specifications	Empty Cell
**Build volume (mm^3)**	130 × 130 × 130
**Supported materials**	ABS, PLA
**Number of extruders**	1
**Heated platform**	Yes
**Minimum layer thickness (mm)**	0.1
**Syringe diameter (mm)**	30
**Nozzle diameter (mm)**	1
**Open Source**	Hardware and software

The component of the syringe unit was designed through SolidWorks™ (Dassault Systèmes, SolidWorks Co., Vélizy-Villacoublay, France) and was developed by Peyman Asghartabar Kashi and was 3D printed from polylactic acid (PLA, eSUN, China) using a homemade extrusion-based 3D printer. We decided to fabricate the components of the syringe unit with another 3D printer than the device we aimed to modify, as this facilitated design optimization and circumvented the need for multiple assembly-disassembly cycles, accelerating the prototyping procedure.

The machine architecture is very simple, where the extrusion head transfers in the XZ plane construction while the platform translates along the Y-axis. The horizontal translation of the head is controlled by the X-axis and the vertical translation allows the increment along the Z-axis. The mechanical structure is minimal

and contains fundamentally two rails along which the building platform is moved, the other two rails for the motion of the extrusion head, and the structure to support the rails.

For the 3D printing process, a snowflake (80 mm diameter, 10 mm height), octopus (80 mm diameter, 40 mm height), and cylindrical (40 mm diameter, 60 mm height) were initially modeled and converted.STL files through the computer-aided design software (SolidWorks™, Dassault Systèmes, SolidWorks Co., Vélizy-Villacoublay, France). Next, the Cura™ slicing software (V. 2.3.1., Ultimaker, Geldermalsen, Netherlands) was used to convert.STL files to G-Code files.

S6. Microstructure comparison in the emulsion with and without soy protein particles

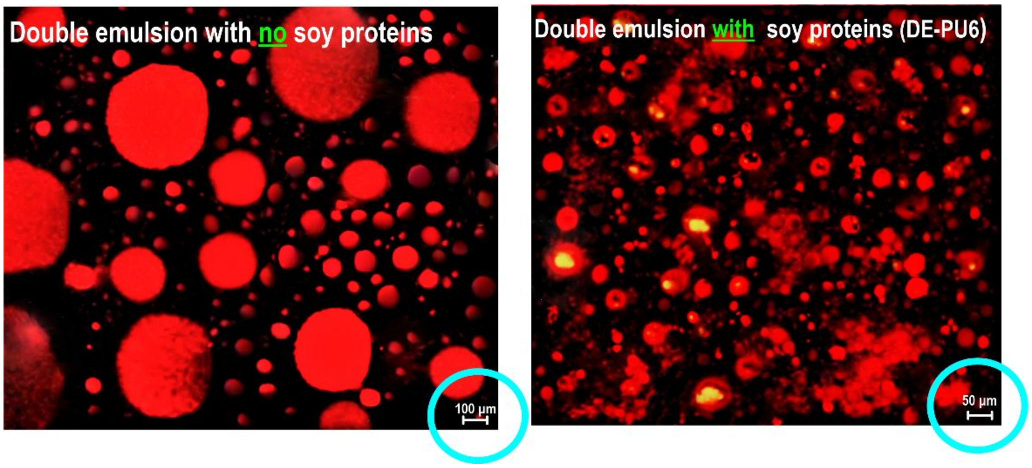

Figure S5. CLSM images of double emulsions with and without soy particles.

S7. Estimating printing maximum shear rate (MSR)

Table S3. Power law indices m and n obtained via curve fitting and the maximum shear rate inside the nozzle for all types of emulsion samples.

Sample	m	n	MSR
DE-PU0	3.89	0.98	483.68
DE-PU2	2.22	0.86	534.50
DE-PU4	29.74	0.63	148.62
DE-PU6	24.86	0.66	133.64
DE-PU8	11.52	0.25	313.60

S8. Cell viability assay: indirect tests

To assess the biocompatibility of the printed scaffolds, a cell viability test was accomplished. Saos-2 (ATCC® HTB-85™) osteoblasts, SH-SY5Y (ATCC® CRL-2266™), and NIH/3T3 cells (ATCC® CRL-1658™) were cultured on three diverse 96 multiwall plates for 24 h to reach confluence through McCoy's 5A Modified Medium (Sigma-Aldrich, Steinheim, Germany) for Saos-2 osteoblasts, and Dulbecco's Modified Eagle Medium (DMEM; Sigma Aldrich, Steinheim, Germany) for SH-SY5Y and NIH/3T3. At the same time, each printing scaffold was soaked in McCoy's 5A (Modified) (1 mL) Media or DMEM (1 mL) each 3D printed scaffold (0.1 g) for 24 h. To assure sterility, each medium was collected and filtered through 0.22 µm filters After 24 h.

Next, culture media were removed from each wall of confluent cells and replaced by a supernatant collected from printing scaffolds. Control was prepared through the normal medium. Supernatants were removed and cell viability tests were carried out through non-fluorescent resazurin after 24 h incubation, converting to the extremely resorufin (red fluorescent dye) through cell metabolism. In brief, 0.2 mg mL^{-1} resazurin solution (100 µL) was incorporated into walls and the cultures were incubated for 60 min at 37 °C. A resazurin solution (0.2 mg mL^{-1}) was prepared through dilution of resazurin working solution (1 mg/mL in phosphate buffered saline-PBS, Merck, Darmstadt, Germany) to DMEM. Following that, fluorescent signals were used by the plate reader VICTOR™ X3 (PerkinElmer, Waltham, MA) at 590 nm emission wavelength and 530 nm excitation wavelength. Cell viabilities were measured as the fraction values in comparison with the control. Three samples for each condition were used and experiments were performed three times.
